# Receptor–Mitochondria Crosstalk in the Kynurenine Metabolic Pathway: Integrating Metabolomics and Clinical Mass Spectrometry

**DOI:** 10.3390/antiox15020261

**Published:** 2026-02-19

**Authors:** László Juhász, Zsolt Galla, Masaru Tanaka, László Vécsei

**Affiliations:** 1 Institute of Surgical Research, Albert Szent-Györgyi Medical School, University of Szeged, H-6725 Szeged, Hungary; juhasz.laszlo.1@med.u-szeged.hu; 2 Department of Pediatrics, Albert Szent-Györgyi Faculty of Medicine, University of Szeged, H-6725 Szeged, Hungary; galla.zsolt@med.u-szeged.hu; 3 HUN-REN-SZTE Neuroscience Research Group, Danube Neuroscience Research Laboratory, Hungarian Research Network, University of Szeged (HUN-REN-SZTE), H-6725 Szeged, Hungary; 4 Department of Neurology, Albert Szent-Györgyi Medical School, University of Szeged, H-6725 Szeged, Hungary

**Keywords:** kynurenic acid (KYNA), mitochondria, tricarboxylic acid (TCA) cycle, nicotinamide adenine dinucleotide (NAD^+^), metabolomics, liquid chromatography–mass spectrometry (LC-MS), receptors, G protein-coupled receptors, aryl hydrocarbon receptor (AhR), N-methyl-D-aspartate (NMDA), mitophagy

## Abstract

Mitochondria govern energy transfer, redox balance, and cell fate. Tryptophan catabolism generates kynurenines (KYNs) that can tune mitochondrial function, with growing evidence that G protein-coupled receptor 35 (GPR35), aryl hydrocarbon receptor (AhR), and N-methyl-D-aspartate receptors (NMDA receptors) link extracellular cues to adenosine 5 prime triphosphate (ATP) maintenance, calcium (Ca^2+^) handling, mitophagy, and inflammasome control. In parallel, quinolinic acid (QA)-driven de novo nicotinamide adenine dinucleotide (NAD^+^) synthesis connects KYN flux to tricarboxylic acid (TCA) cycle activity and sirtuin programs across tissues. Key gaps remain: receptor pharmacology is rarely integrated with NAD^+^ economics and respiration, and clinical workflows still lack single-run assays that quantify both kynurenine and TCA nodes. We therefore integrate receptor proximal signaling, QA-driven NAD^+^ supply, and unified liquid chromatography–mass spectrometry (LC-MS) measurement into one translational framework spanning kynurenic acid (KYNA), KYN, 3-hydroxykynurenine (3-HK), and QA, using mitochondrial endpoints as the common readout. We synthesize evidence for mitochondrial GPR35 signaling that preserves ATP, AhR programs that tune oxidative defenses and mitophagy, and NMDA receptor antagonism that limits excitotoxic stress. These mechanisms are linked to QA-dependent NAD^+^ biogenesis and alpha ketoglutarate control points, then aligned with chromatography and ionization choices suited to routine LC-MS workflows. This receptor to organelle framework couples KYN flux to respiratory control and provides a practical roadmap for standardized single-run LC-MS panels. It can strengthen target validation in ischemia, neurodegeneration, psychiatry, and oncology while improving biomarker qualification through harmonized analytics and decision-grade readouts.

## 1. Introduction

Mitochondria choreograph energy flux, redox poise, and fate decisions through tightly coupled metabolism, signaling, and quality control (QC) [1,2]. Nuclear factor erythroid 2-related factor 2 (Nrf2) and nuclear respiratory factor 1 (Nrf1) align bioenergetics with antioxidant defenses, tuning respiration, detoxification, and ROS setpoints to prevent maladaptive stress responses [3,4]. Proton leak via uncoupling proteins subtly tempers superoxide, reshaping signaling without collapsing adenosine 5′-triphosphate (ATP) supply [5,6]. Dynamic cycles of fission, fusion, biogenesis, and mitophagy purge damage and license apoptosis or survival, thereby preserving tissue function across development and aging [1,7,8]. In stem and neuronal lineages, mitochondrial metabolites and ROS act as instructive cues that program transcription and differentiation while guarding viability [2,9,10]. These convergent circuits constitute a master key for health and disease [11,12].

Tryptophan (Trp) catabolism feeds the kynurenine (KYN) metabolic pathway, yielding kynurenic acid (KYNA) that operates as a pleiotropic signal aligning mitochondrial respiration, redox poise, and cellular metabolism [13,14,15]. KYNA engages G protein–coupled receptor 35 (GPR35) and AMP-activated protein kinase (AMPK) to modulate bioenergetics, thermogenesis, and lipid handling across adipose and muscle, with exercise-driven KYN aminotransferases (KATs) activity boosting KYNA output and efficiency [16,17,18]. Genetic or pharmacologic perturbation of KAT enzymes reveals KYNA’s necessity for ATP synthesis and mitochondrial stability in brain and peripheral tissues [13,19,20,21]. At stress frontiers, KYNA preserves ATP, curbs mtROS, and licenses mitophagy, thereby limiting inflammasome activation and ischemic injury [21,22,23,24]. Pathway flux also supports NAD^+^ economy and neuroprotection, linking immune tone to mitochondrial longevity and disease modification [14,25,26,27,28,29,30].

KYNA coordinates receptor signaling that feeds directly into mitochondrial control. As outlined in Section 2, KYNA GPR35 signaling can interface with ATPIF1 to limit ATP synthase reverse activity and protect inner membrane function, so downstream disease effects are interpreted here as readouts of that conserved energy preservation module [22,31]. GPR35 signaling restrains calcium (Ca^2+^) mobilization, limits NLRP3 activation, and enables autophagic disposal of inflammasomes, linking immunity to mitochondrial QC [32,33]. As an AhR ligand, it reprograms redox and apoptotic set points across neural and cardiovascular contexts [34,35]. Antagonism at *N*-methyl-*D*-aspartate (NMDA) receptors and mitochondrial nicotinic acetylcholine receptors containing α7 subunits (α7nAChR) rewires excitatory drive and metabolic coupling, tuning respiration and protecting tissue function [36,37]. Additional endogenous ligands at GPR35 add complexity to this regulatory axis [38,39].

Trp degradation through the KYN metabolism supplies de novo nicotinamide adenine dinucleotide (NAD^+^), the redox currency that feeds the tricarboxylic acid cycle (TCA) and the electron transport chain, thereby tuning respiration at its core [14,40]. Flux through this pathway sets mitochondrial NAD^+^/NADH ratios and ROS thresholds via redox-active intermediates, stabilizing oxidative phosphorylation and ATP output [15,41]. Immune and tissue contexts reveal causality: macrophages require pathway-derived NAD^+^ for oxidative metabolism, while ischemia–reperfusion diverts flux and collapses antioxidant capacity until NAD^+^ is restored [42,43]. Enzyme control points are actionable. ACMSD inhibition elevates NAD^+^; kynurenine 3-monooxygenase (KMO) modulation redirects carbon to sustain TCA activity; and pathway blockade diminishes SIRT1 signaling and viability [44,45]. These circuitries extend to microbiota, cardio-metabolic risk, T-cell bioenergetics, cancer, and aging [45,46,47,48,49].

The KYN metabolism links inflammation to mitochondrial bioenergetics, and its clinical footprint spans neurology, psychiatry, ischemic injury, metabolism, and cancer [26,50]. In neurodegeneration, skewed production of neurotoxic versus protective metabolites accelerates oxidative stress, excitotoxicity, and decline, while enzyme targeting can tilt the balance toward resilience [51,52,53]. Psychiatric syndromes display immune-driven pathway activation with measurable biomarker shifts and actionable enzymatic nodes [50,54,55,56,57,58,59]. Cardiovascular and systemic contexts reveal redox and immune dysregulation that worsens tissue injury and aligns with ischemic vulnerability [60,61]. Metabolic disease reflects chronic low-grade inflammation that routes Trp away from homeostasis [26,62]. Tumors exploit pathway-derived NAD^+^ and immunosuppression, creating therapeutic entry points under active clinical evaluation [63,64].

Capturing KYN metabolites and tricarboxylic acid intermediates in the same clinical sample remains a moving target [65,66,67]. Targeted LC-MS workflows for KYN species vary widely in extraction, chromatography, and calibration, and no protocol robustly spans all key metabolites or matrices [65,66,68]. Matrix effects, polarity extremes, and poor chromatographic behavior of compounds such as quinolinic acid (QA) confound accuracy and comparability [65,69]. Parallel LC-MS assays for TCA intermediates add further hurdles due to instability and matrix-dependent losses [65,70]. Alternative readouts help but fragment the picture: voltammetry and immunostrips deliver speed at the cost of scope, while capillary electrochromatography trades coverage for protracted runs [71,72]. Clinically useful panels will demand matrix-specific preparation, isotope-labeled standards, and harmonized cross-platform validation [65,73].

Current evidence offers vivid mechanistic snapshots, yet the mosaic remains disjointed across species, cell types, and measurement scales [21,55]. Elegant studies in lupus-prone T cells link Rab4A trafficking to mitophagy, cluster of differentiation 98 (CD98), and KYN-sensitive mTOR, but insights are constrained by model specificity and temporal windows [47,74]. Clinical syntheses in depression expose state-dependent metabolite signatures alongside striking heterogeneity in cohorts and methods [50,75]. Cross-phyla work in Lymnaea underscores evolutionary conservation while complicating translation to humans [76,77]. Analytical platforms further splinter datasets, with electrochemical and chromatographic approaches optimized for different matrices and targets [78,79]. Reviews connecting the pathway to NAD^+^ and aging highlight gaps between molecular flux, organelle dynamics, and outcomes that matter clinically [50,77,80,81,82].

**Figure 1:**
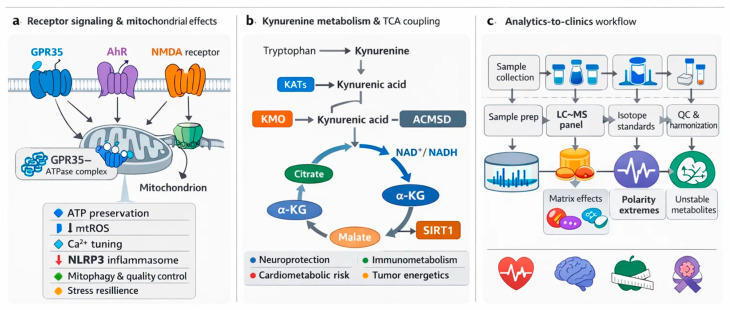
KYN pathway metabolites as mitochondrial gatekeepers: receptor signaling, QA-driven NAD^+^ coupling, and an analytics-to-clinics workflow. (**a**) Receptor landscape and mitochondrial endpoints linked to KYNA, highlighting GPR35, AhR, NMDA-R, and α7nAChR signaling. (**b**) KYN pathway to TCA coupling, showing how branch points such as KATs, KMO, and ACMSD route flux toward KYNA signaling versus QA-to-NAD^+^ supply and shape mitochondrial redox state and respiratory throughput. (**c**) Analytics-to-clinics workflow summarizing LC-MS panel design, matrix and stability constraints, and QC steps needed for reproducible clinical measurement. Mechanistic and metabolic links are developed in Section 2 and Section 3, while measurement and translation are expanded in Section 4 and Section 5. ACMSD, alpha-amino-beta-carboxymuconate-epsilon-semialdehyde decarboxylase; AhR, aryl hydrocarbon receptor; α7nAChR, alpha-7 nicotinic acetylcholine receptor; GPR35, G protein-coupled receptor 35; KATs, kynurenine aminotransferases; KMO, kynurenine 3-monooxygenase; KYN, kynurenine; KYNA, kynurenic acid; LC-MS, liquid chromatography–mass spectrometry; NAD^+^, nicotinamide adenine dinucleotide; NMDA-R, N-methyl-D-aspartate receptor; QA, quinolinic acid; QC, quality control; TCA, tricarboxylic acid.

Existing reviews have typically advanced one axis at a time. Neurology- and psychiatry-focused syntheses emphasize pathway shifts and biomarker associations, often framing KYNA and QA as neuroprotective versus neurotoxic signals without fully resolving how extracellular receptor engagement is translated into organelle level control of ATP, Ca^2+^, mitophagy, and inflammasome tone [83,84,85]. Aging- and NAD^+^-centered reviews clarify the logic of quinolinate-driven de novo NAD^+^ supply, sirtuin programs, and redox buffering, yet they rarely connect these metabolic steps to receptor pharmacology or to clinical-grade analytics that can quantify both KYN and TCA nodes in a single workflow [86,87]. Immunometabolism and oncology-oriented discussions outline IDO- and TDO-driven tolerance circuits and AhR-dependent immune rewiring, but mitochondrial mechanism is often treated as background physiology rather than a defined causal layer [88,89] (Figure 1).

This review contributes three integration moves that are not usually delivered together. First, it uses a receptor to mitochondria framework that treats GPR35 trafficking, AhR programs, and NMDA-linked Ca^2+^ gating as proximal controllers of mitochondrial decision points, rather than downstream correlates [22,90]. Second, it formalizes a QA to NAD^+^ to TCA scaffold that links de novo NAD^+^ supply to respiratory control and redox set points, and then identifies actionable checkpoints where flux and signaling intersect [91] (Figure 1). Third, it connects analytics to clinics by evaluating LC-MS designs that can co-quantify KYN and TCA intermediates in one run, and by outlining quality control and harmonization steps that make multi-cohort translation realistic (Table 1).

Despite striking mechanistic vignettes, four gaps impede synthesis. First, temporal dynamics remain under-sampled: circadian, acute, and chronic windows yield non-overlapping readouts that are rarely integrated [98]. Second, signaling is compartment-specific across tissues, cell types, and subcellular locales, complicating translation from regionally restricted or model-bound observations [99]. Third, causal links from receptors such as AhR or GPR35 to mitochondrial remodeling are inferred more often than demonstrated longitudinally in vivo [22]. Fourth, multi-analyte assays lack harmonization across matrices, throttling cross-study comparability and biomarker qualification [66]. Addressing these deficits will require time-resolved, compartment-aware designs that couple receptor activation to mitochondrial endpoints while deploying standardized, multiplexed metabolomic and signaling panels across preclinical and clinical cohorts [81,98].

Objectives are fourfold. First, chart receptor-specific mitochondrial actions of KYNA by resolving GPR35-dependent Ca^2+^ control, ATP preservation, and inflammasome restraint, and by contrasting AhR-driven stress programs and synaptic α7nAChR modulation. Second, delineate pathway–cycle crosstalk by linking enzyme localization and NAD^+^ biogenesis to respiratory control and organelle dynamics across tissues and time. Third, appraise integrated analytics that fuse targeted LC-MS panels with multi-omics, isotope tracing, and trafficking readouts to capture mechanism and flux in matched samples. Finally, synthesize translational implications in ischemic protection, neurodegeneration, cancer immunity, and network-level metabolic resilience to guide trial design and biomarker qualification.

Bridging correlation to cure requires two pillars. First, standardized, validated quantification across matrices and cohorts so biomarker signals mean the same thing in every lab [100,101]. Second, causal mechanistic experiments that link receptor and enzyme perturbations to mitochondrial dynamics and clinical outcomes [102]. Harmonized LC-MS/MS panels and fit-for-purpose QC will enable longitudinal, multi-analyte readouts in neurology, psychiatry, cardiometabolic disease, and oncology, converting meta-analytic heterogeneity into actionable thresholds [103]. Interventional studies that pair enzyme inhibition or pathway rerouting with mitochondrial endpoints can validate target engagement and refine patient selection [104]. The sections that follow track these objectives in order, moving from receptor to mitochondria mechanisms, to QA-linked NAD^+^ and TCA control, to assay design choices, and finally to translational decision points (Figure 1).

## 2. Receptor- and Metabolite-Driven Mitochondrial Regulation Within the Kynurenine (KYN) Pathway

KYNA sits at a neat signaling crossroads, but it is not the only KYN that can steer mitochondrial behavior in a mechanistically meaningful way. KYN itself can act through the aryl hydrocarbon receptor (AhR)-biased programs that reshape oxidative defenses and tune mitophagy, so the pathway branch point already carries receptor-linked mitochondrial consequences. Step downstream and the chemistry sharpens. 3-Hydroxykynurenine (3-HK) can tip redox balance toward oxidative stress, while QA can couple de novo NAD^+^ supply to bioenergetic control yet also intensify excitotoxic pressure when it accumulates. Taken together, these metabolites create a layered control system in which receptor signaling and metabolite driven stress converge on shared endpoints such as respiration, reactive oxidative species handling, Ca^2+^ homeostasis, and mitochondrial quality control, with tissue specific fingerprints across brain, immune cells, and tumors (Table 2).

### 2.1. Kynurenic Acid (KYNA) and G Protein-Coupled Receptor 35 (GPR35): Energy Homeostasis and Ischemic Protection

GPR35 has emerged as a regulator of metabolic stress responses [105,106,107,108,109]. It is ex-pressed in the gastrointestinal tract, immune cells, central nervous system, and heart, with expression upregulated in pathological conditions including ischemia–reperfusion injury, hypoxia, stroke, and heart failure [110,111,112]. Although GPR35 is associated with cytoprotection, evidence remains inconsistent regarding whether receptor activation or inhibition is beneficial [110,111,113,114]. Furthermore, a GPR35-dependent gut–microbe–brain metabolic axis has been identified, linking receptor activity to neuroimmune regulation and depressive-like behavior [115,116].

GPR35 interacts with several G protein families, enabling the regulation of diverse intracellular pathways with both pro- and anti-inflammatory effects [114,117,118,119]. Coupling to Gαi/o suppresses adenylate cyclase, decreases cyclic adenosine monophosphate (cAMP) levels [120], and reduces extracellular signal-regulated kinase (ERK) activity [121], which can limit extracellular signal-regulated kinase (ERK)-driven pro-inflammatory transcription [117]. Conversely, Gβγ subunits released from Gαi/o activate PLCβ, driving phosphoinositide hydrolysis, phosphoinositide 3-kinase (PI3K)/protein kinase B (AKT) signaling, and nuclear factor kappa-light-chain-enhancer of activated b cells (NF-κB) activation, thereby promoting inflammatory gene expression [117]. Interaction with Gα12/13 stimulates ras homolog family protein (Rho)-dependent cytoskeletal remodeling, enhancing immune cell chemotaxis and reinforcing pro-inflammatory signaling. In addition, Gαq coupling exerts dual actions: it restricts PI3K-mediated AKT activation while facilitating ERK signaling through a PLCβ/Ca^2+^/Src pathway [122]. These diverse and occasionally opposing mechanisms underscore the context-dependent role of GPR35 in coordinating G protein signaling and regulating mitochondrial responses [32,123,124,125]. Activation of these receptors has also been described to contribute to organellar damage via calpain-mediated proteolysis under various pathophysiological conditions. For example, Ca^2+^ overload activates calpains, which translocate to intracellular organelles, degrade target proteins, destabilize nuclear, lysosomal, and mitochondrial membranes, and release cathepsins and pro-apoptotic factors, ultimately leading to cell death [126]. Evidence implicates calpain-1 and calpain-2 in mitochondrial damage following cardiac ischemia/reperfusion [113,127,128]. Notably, GPR35 is strongly upregulated after myocardial ischemia, and its inhibition attenuates ROS production, reduces mitochondrial apoptosis, and preserves contractile function [129,130]. Consistently, blockade of GPR35 downregulates calpain-1 and calpain-2 expression and activity, attenuating calpain-mediated mitochondrial injury. The detrimental effects of calpain-driven proteolysis affect multiple mitochondrial sites, including increasing mitochondrial membrane permeability, inducing cytochrome c release, and initiating apoptosis [131]. In addition, calpain-1 can cleave the ATP synthase α-subunit, reducing ATP production and exacerbating oxidative stress [131].

Wyant and colleagues (2022) demonstrated that KYNA exerts cardioprotective effects during ischemia/reperfusion by acting on GPR35 [22]. Their study identified GPR35 as both necessary and sufficient for mediating KYNA-induced ischemic protection, a process tightly coupled to mitochondrial remodeling. Upon ligand binding, GPR35 activates Gi- and G12/13-dependent signaling cascades and translocates to the outer mitochondrial membrane, where it associates, likely indirectly, with ATP synthase inhibitory factor subunit 1 (ATPIF1) [118,132]. Through this interaction, activated GPR35 promotes ATP synthase dimerization and modulates oxidative phosphorylation to preserve cellular ATP content and maintain energy homeostasis under ischemic stress [133,134,135]. ATP synthase normally generates ATP from the proton gradient; however, during ischemia, it can reverse and hydrolyze ATP, resulting in energy loss and mitochondrial dysfunction [136,137,138]. ATPIF1 prevents this reverse mode without affecting ATP synthesis [110]. By stabilizing ATP synthase dimers, ATPIF1 also supports cristae integrity and prevents mitochondrial permeability transition pore (mPTP) opening [22,139]. In more detail, phosphorylation of ATPIF1 deactivates the protein, thereby permitting ATP hydrolysis, whereas dephosphorylation at Ser39 activates ATPIF1 and suppresses ATPase activity [22,140]. Mitochondrial GPR35 signaling dampens cAMP production by inhibiting adenylyl cyclase, thereby reducing PKA-mediated phosphorylation of ATPIF1 and maintaining it in its active, dephosphorylated state [22]. Collectively, these findings delineate a mitochondrial GPR35 in coordinating energy conservation and structural stability.

The mitochondrial membrane potential (ΔΨm) serves as the primary driving force for ATP synthesis, generated by the proton gradient across the inner mitochondrial membrane [141,142,143]. A decrease in ΔΨm weakens the proton motive force, leading to diminished or even halted ATP production. Sustained depolarization of the mitochondrial membrane results in cellular energy depletion, metabolic disturbances, and ultimately, cell death [144,145]. Preservation of ATP levels under ischemic conditions is therefore closely dependent on maintaining ΔΨm, which is essential for cell viability and the physiological function of organs [145]. A direct link between GPR35 modulation and alterations in ΔΨm has also been demonstrated under pathological conditions. Specifically, inhibition of GPR35 was shown to mitigate mitochondrial dysfunction not only by enhancing oxidative phosphorylation but also by preserving ΔΨm [113]. In neonatal murine ventricular myocytes (NMVMs), JC-1 assays revealed a higher ΔΨm under ischemic or anoxic stress when GPR35 was inhibited compared to control conditions, indicating a protective mitochondrial effect [113].

Beyond its well-established role in ischemic protection, accumulating evidence indicates that KYNA–GPR35 signaling constitutes a critical regulatory axis in systemic energy homeostasis [16,146,147], particularly in the regulation of lipid catabolism [17]. Following three days of KYNA administration (a single daily intraperitoneal dose of 5 mg/kg body weight) in C57BL/6J mice, increased oxygen consumption, carbon dioxide production, and heat generation were observed, indicating enhanced metabolic activity and energy expenditure [16]. Moreover, KYNA administered on consecutive days significantly reduced white adipose tissue mass, including both inguinal and visceral (epididymal) depots, without exerting any measurable effect on brown adipose tissue mass. In adipose tissue, activation of GPR35 induces thermogenic and anti-inflammatory transcriptional programs, and thereby mitigates high-fat diet-induced adiposity while simultaneously improving glucose tolerance [16]. At the molecular level, this pathway upregulates PGC-1α expression and enhances mitochondrial oxidative capacity, thereby promoting mitochondrial biogenesis [16]. The anti-inflammatory mechanism involves KYNA signaling, which increases the expression of type 2 cytokines such as IL-4, IL-10, IL-13, and IL-33, while reducing pro-inflammatory markers such as TNFα [16,148]. This cytokine shift promotes a type 2 immune environment, supporting the resolution of inflammation and improving insulin sensitivity [17]. The KYNA–Gpr35 pathway has been shown to enhance the presence and activity of regulatory T cells (Tregs) and type 2 innate lymphoid cells (ILC2s), thereby contributing to anti-inflammatory signaling and adipose tissue beiging [16].

The KYNA–Gpr35 axis has emerged as a critical regulator of adipose tissue metabolism and inflammation, with considerable therapeutic relevance [16,17,34]. KYNA exerts a dual modulatory effect on metabolic efficiency and immune homeostasis by promoting adipose tissue beiging and enhancing mitochondrial oxidative capacity to support thermogenesis [16]. In parallel, KYNA modulates the inflammatory milieu, directing the immune balance toward an anti-inflammatory phenotype [17,147,149]. These coordinated actions integrate metabolic and immunoregulatory mechanisms, suggesting that pharmacological activation of Gpr35 may constitute a promising therapeutic approach to augment systemic energy expenditure and mitigate metabolic disorders associated with chronic low-grade inflammation and disrupted energy balance, including obesity, type 2 diabetes, and metabolic syndrome [16].

### 2.2. Kynurenic Acid (KYNA) and Aryl Hydrocarbon Receptor (AhR): Mitophagy and Organelle Quality Control (QC)

The AhR functions as a ligand-activated transcription factor that remains localized in the cytoplasm under basal conditions [150,151,152]. Upon ligand binding, AhR undergoes conformational changes that promote its nuclear translocation and the transcriptional regulation of a broad array of genes involved in cellular homeostasis [95,153,154,155]. AhR is well recognized for its central role in detecting xenobiotics and regulating their metabolism through cytochrome P450 enzymes (e.g., CYP1A1, CYP1A2, and CYP1B1) [156,157]. Moreover, increasing evidence indicates that AhR participates in a wide range of physiological processes, including immune regulation and embryogenesis [158,159,160]. However, its contribution to mitochondrial regulation in association with the Trp-KYN pathway has only recently come to light.

The first study identifying KYNA as an endogenous agonist of AhR was reported in the early 2010s [161,162]. Like KYNA, xanthurenic acid (XA)—another metabolite of the Trp–KYN pathway—has been shown to function as a ligand that can activate the AhR at physiologically relevant concentrations [161,163]. Recent evidence has demonstrated that AhR directly contributes to hepatic energy preservation by modulating mitophagy [155]. Mitophagy is a selective form of autophagy occurring within lysosomes and is responsible for the removal of damaged mitochondria [164,165,166,167,168]. This process reduces mitochondrial ROS overproduction, limits inflammasome activation, decreases apoptosis, maintains proper ATP synthesis, and promotes mitochondrial turnover [169,170,171]. In both AhR knockout mice and hepatocyte models, the loss of AhR expression resulted in impaired mitochondrial respiration, decreased substrate utilization, and dysregulation of mitochondria-associated gene networks [155,171]. Under pathophysiological conditions, a study conducted in intestinal porcine enterocytes (IPEC-J2 cells) further illustrated that activation of the AhR by Trp ameliorates lipopolysaccharide (LPS)-induced inflammatory responses [172,173]. This work also provided mechanistic insight into the interplay between AhR activation and mitochondrial QC by demonstrating that AhR functions as a direct transcriptional activator of PINK1 [155,172,174].

Mitophagy, in addition to being initiated through the ubiquitin-dependent PINK1–Parkin pathway, can also occur via receptor-mediated mechanisms involving BCL2-interacting protein 3 (BNIP3) and NIX [175,176,177,178,179]. In this pathway, LC3 binds to these receptors through its LC3-interacting region (LIR), ensuring the targeted recruitment of autophagosomes to mitochondria and enabling selective mitochondrial degradation [180]. Fasting strongly induced Bnip3 expression in livers obtained from wild-type samples, whereas this response was completely absent in AhR-deficient mice [155]. Activation of AhR by its endogenous ligand KYN significantly increased Bnip3 mRNA and protein levels in primary hepatocytes and AML12 cells [155]. Moreover, inhibition of AhR elevated mitochondrial ROS production—an effect entirely reversed by BNIP3 overexpression—and decreased LC3A expression [155]. Together, these results indicate that AhR plays a crucial role in regulating receptor-mediated mitophagy in the liver.

Excessive mitochondrial ROS promote mitochondrial dysfunction [181,182,183], a mechanism previously linked to AhR activation and AhR-dependent ROS generation induced by dioxins [184]. One potential source of these ROS is the accumulation of damaged or dysfunctional mitochondria, highlighting the importance of their removal for proper cellular function, including ATP production [185,186]. Physiological AhR activity appears to play a significant role in controlling mitochondrial stress responses, as its inhibition or loss has been associated with ROS imbalance and disturbance in oxidative metabolism [184,187]. In the liver, results suggests that AhR contributes to hepatic metabolic adaptation by maintaining mitochondrial efficiency and redox balance under nutrient, environmental, or hypoxic stress. Activation of AhR supports oxidative metabolism and preserves energy homeostasis, whereas its inhibition disrupts metabolic balance and promotes ROS accumulation, which can reduce cell viability [155].

The AhR plays a cellular-context–dependent role in mitochondrial biology, mediating both protective and deleterious outcomes. Under physiological conditions, AhR supports mitochondrial homeostasis: in hepatocytes, AhR loss impairs mitophagy, increases mitochondrial ROS, and reduces electron transport system function [155]. Similarly, in human melanocytes following H_2_O_2_-induced oxidative injury, AhR activation promotes mitochondrial biogenesis, mtDNA synthesis, and ATP production via NRF1 upregulation [188]. Conversely, toxic or xenobiotic ligands, such as PM2.5 or elevated KYN, trigger AhR-mediated mtROS production, mPTP opening, and ΔΨm collapse, leading to apoptosis in mouse neuronal cells and zebrafish heart [189,190]. These findings raise key questions: How do cell type, tissue context, and metabolic state dictate whether AhR signaling is protective or deleterious? How do ligand characteristics, dose, and exposure time affect mitochondrial outcomes? Resolving these issues is critical for understanding AhR’s divergent role in mitochondrial physiology.

### 2.3. Kynurenic Acid (KYNA) and N-Methyl-D-Aspartate (NMDA) Receptors: Ca^2+^ Regulation and Excitotoxicity

**Figure 2:**
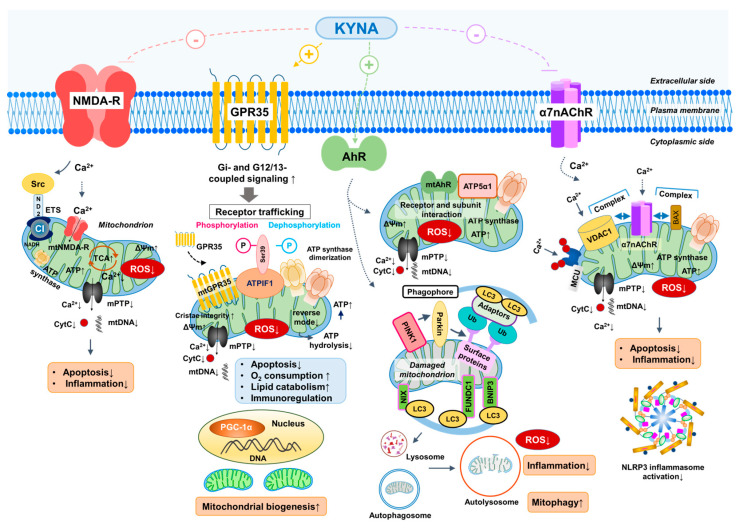
KYNA-linked receptor signaling converges on mitochondrial bioenergetics, redox balance, calcium handling, and quality control during metabolic or ischemic stress. GPR35-coupled energy preservation module that supports ATP maintenance and inner membrane stability. AhR-linked redox tuning and mitophagy programs that promote selective clearance of damaged mitochondria. NMDA-R linked calcium entry control that lowers excitotoxic pressure and reduces pore sensitivity. Mitochondrial alpha7nAChR-linked modulation of VDAC-associated calcium flux and respiratory ROS output. Detailed mechanisms are described in Section 2, while QA to NAD^+^ and TCA coupling is developed in Section 3. AhR, aryl hydrocarbon receptor; alpha7nAChR, alpha7 nicotinic acetylcholine receptor; ATP, adenosine triphosphate; ETC, electron transport chain; GPR35, G protein-coupled receptor 35; KYNA, kynurenic acid; mitoKATP, mitochondrial ATP sensitive potassium channel; NAD^+^, nicotinamide adenine dinucleotide; NMDA-R, N methyl D aspartate receptor; QA, quinolinic acid; ROS, reactive oxygen species; TCA, tricarboxylic acid cycle; VDAC, voltage-dependent anion channel.

NMDA-Rs are glutamate receptors and ligand-gated channels, widely recognized for their roles in synaptic signaling, Ca^2+^ regulation, and excitotoxicity. Although they are predominantly expressed in the central nervous system, receptor subunits have also been detected in peripheral organs, including the heart, stomach, and intestine. Emerging evidence indicates that NMDA-R localization is not restricted to the plasma membrane, as these receptors have also been identified in the inner mitochondrial membrane, suggesting a potential role in regulating mitochondrial function (Figure 2).

KYNA acts as an NMDA-R antagonist by exerting its inhibitory effect at the glycine co-agonist site of the receptor [41,191,192], resulting in pleiotropic protective effects that include the modulation of mitochondrial function through this mechanism [13,193,194].

NMDA-R overdrive elevates cytosolic Ca^2+^ and can push mitochondria past their buffering range. When Ca^2+^ loading coincides with oxidative pressure, pore sensitivity rises, ΔΨm drops, and apoptosis-linked signaling accelerates. In this context, KYNA antagonism at NMDA-R functions less like a single-target trick and more like a gatekeeper that lowers Ca^2+^ entry, which reduces downstream mitochondrial destabilization and inflammatory spillover.

By limiting NMDA-R-mediated Ca^2+^ influx, KYNA may indirectly reduce the probability of mPTP opening, thereby preventing the release of both cytochrome c and mtDNA into the cytosol [13,195]. This dual action not only attenuates pro-apoptotic signaling but also mitigates inflammatory processes associated with mitochondrial damage, emphasizing the potential cyto- and mitoprotective roles of KYNA. Recent studies further support the anti-apoptotic effects linked to its mitoprotective actions, although these mechanisms are not solely mediated by NMDA-Rs. In H9c2 cells and primary rat cardiomyocytes exposed to simulated ischemia/reperfusion [31,125], KYNA exerted a dose-dependent effect on cell viability, with the most effective concentration being 64 µM [125]. Specifically, KYNA attenuated intramitochondrial Ca^2+^ accumulation, reduced ROS generation, and alleviated alterations in mitochondrial network architecture following simulated ischemia/reperfusion. Moreover, apoptosis markers such as caspase-3/7 and BAX (a pro-apoptotic modulator) were reduced, while the expression of the anti-apoptotic protein Bcl-XL was increased following KYNA treatment [125]. Consistent with these mitoprotective and anti-apoptotic effects, a reduction of neuronal apoptosis in microglial cultures via attenuation of CXCL10 expression by KYNA and its analogue (SZR-104) cannot be ruled out [196].

NMDA-Rs are not limited to the plasma membrane but have also been detected in mitochondria [197]. The presence of NR1 and NR2B subunits, together with GABAA (alpha-6) and GABAB (R2) receptors, has been reported in rat heart mitochondria. In addition, extensive NR2a subunit immunoreactivity was observed on hippocampal mitochondria using immunogold electron microscopy [198]. Although the precise role of these receptors within mitochondria remains unclear, they are hypothesized to regulate Ca^2+^ fluxes, modulate ROS production, and contribute to metabolic adaptation under hypoxic or ischemic conditions [198,199]. An additional layer of regulation may involve direct interactions between NMDA-R subunits and mitochondrial complex I components, such as ND2, mediated via Src adaptor proteins [200,201]. This receptor–complex I crosstalk establishes a direct molecular link between receptor activity and mitochondrial energy metabolism, providing a mechanistic framework for how glutamatergic signaling influences mitochondrial bioenergetics. Collectively, these findings suggest that mitochondrial NMDA-Rs may serve as critical modulators of organelle function, integrating cellular signaling with energy generation and oxidative stress responses, with potential implications for pathophysiological conditions such as ischemia, hypoxia, and neurodegeneration.

Taken together, these findings suggest that KYNA exerts multifaceted, receptor-mediated effects on mitochondria. Through interactions with GPR35, AhR, and NMDA-Rs, KYNA helps preserve energy homeostasis, supports mitochondrial quality control, and protects against Ca^2+^-induced mitochondrial dysfunction—highlighting its therapeutic potential in conditions associated with mitochondrial stress or impairment [30,202,203,204,205]. Mitochondrial dysfunction has been implicated in various pathologies, including neurodegenerative diseases such as Alzheimer’s, Huntington’s, and Parkinson’s diseases, as well as psychiatric disorders linked to mood disturbances, such as bipolar depression and migraine [13,41,206,207,208,209,210]. Therefore, maintaining mitochondrial homeostasis appears to be a promising therapeutic strategy for these conditions [174].

Future studies should investigate the role of mitochondrial NMDA-Rs and their modulation by KYNA and its analogues, as it remains unclear whether these channels are sensitive to conventional NMDA-R inhibitors. Likewise, the potential for targeting mitochondrial GPR35 and AhR receptors under stress conditions warrants further exploration, particularly regarding how their trafficking influences mitochondrial membrane dynamics and transport processes. Elucidating these mechanisms may provide crucial insights into how KYNA and its signaling pathways regulate mitochondrial function and could reveal novel therapeutic targets for diseases characterized by impaired bioenergetics or oxidative stress.

### 2.4. Nicotinic Acetylcholine Receptors (α7nAChR)

α7nAChRs, a type of ligand-gated ion channel previously thought to be localized only to the plasma membrane, are also present in the outer mitochondrial membrane [211]. Electron microscopy and binding assays (α-bungarotoxin, α-cobratoxin) have confirmed their presence in this organelle; however, their precise role remains to be characterized.

These α7nAChRs are thought to interact with voltage-dependent anion channels (VDAC1) (Figure 2). Kalashnyk et al. confirmed this interaction, identifying α7 nAChR–Bax and α7 nAChR–VDAC1 complexes in mitochondria isolated from human glioblastoma astrocytoma cells [212]. Pharmacological studies demonstrated that inhibition of α7nAChRs using antagonists such as methyllycaconitine or α7-specific antibodies suppressed mitochondrial cytochrome c release, whereas stimulation with the receptor agonist PNU 282987 enhanced it in isolated mouse liver mitochondria [213]. Furthermore, mitochondrial ROS production was reduced following both receptor inhibition (methyllycaconitine) and stimulation with acetylcholine [213]. The α7nAChRs exhibit relatively high permeability to Ca^2+^ ions, which can influence mitochondrial function by stimulating Ca^2+^ influx and efflux through the mitochondrial Ca^2+^ uniporter and mPTPs [214]. In addition, modulation of various kinase pathways—including PI3K/Akt, Ca^2+^–calmodulin, and Src kinase-dependent signaling—appears to influence mPTP activity via these receptors, thereby supporting the OXPHOS machinery and maintaining cellular energy production [213].

Surprisingly, KYNA has also been reported to inhibit α7nAChRs; however, these findings remain controversial [37], as several research groups have failed to reproduce the original results. One possible explanation for these discrepancies may arise from the distinct pharmacological profiles of the kynurenate analogues most frequently used, such as 7-chloro-kynurenic acid (7-CKA) and 5,7-dichloro-kynurenic acid, which act at different sites on the NMDA-R. A similar mechanism might account for the observed inconsistencies in the context of α7nAChRs as well.

It remains an important question, given conflicting experimental data [215], whether modulation of the α7nAChR—by inhibition or activation—can provide sustained neuroprotection and anti-inflammatory effects, and how these mechanisms interact with each other. At the same time, the potential role of mitochondrial α7nAChRs in mediating the cellular and mitoprotective effects of KYNA or its synthetic analogues has yet to be comprehensively characterized and requires further investigation to bridge the gap between cellular and subcellular mechanisms.

### 2.5. Other Potential Kynurenic Acid (KYNA)-Sensitive Sites

Beyond the canonical receptor set, KYNA likely engages a wider mitochondrial “sensing” network. Additional receptors and redox-tunable targets—especially those shaping ion homeostasis and respiratory control—could help explain KYNA-linked phenotypes in ischemia and inflammation, and may open testable directions for neurodegenerative and psychiatric disease mechanisms.

Mitochondrial ATP-sensitive potassium (mitoKATP) channels are crucial mediators of cardioprotection induced by ischemic preconditioning and neuroprotection following cerebral ischemia–reperfusion. Pharmacological blockade of these channels with 5-hydroxydecanoate abolishes cardioprotective effects, whereas activation with the mitoKATP opener diazoxide confers neuroprotection [216,217]. Mechanistically, mitoKATP channel opening attenuates mitochondrial Ca^2+^ overload and delays or inhibits the opening of mPTP. Given that ROS and elevated Ca^2+^ are major triggers of mPTP opening, and that GPR35 modulates Ca^2+^ flux while ROS can induce mitoKATP opening and upregulate GPR35 [218], a potential crosstalk between these two mitochondrial receptors—both of which influence mPTP function—may underlie coordinated cytoprotective signaling in these disease conditions.

Mitochondrial ROS originate from 16 distinct redox sites within the organelle, with Complexes I and III of the electron transport system representing the principal ROS generators [219,220]. Redox-sensitive cysteine residues in Complex I subunits, such as NDUFS1, NDUFS2, and ND3, fine-tune ROS generation (superoxide and H_2_O_2_) by acting as redox switches. In contrast to excessive ROS production, transient Complex I-derived ROS induction enhances stress tolerance and lifespan in *C. elegans* [221]. KYNA, which scavenges hydroxyl radicals and superoxide [222], may reduce mitochondrial ROS at Complexes I and III by preserving electron transport integrity.

### 2.6. Kynurenine (KYN) as an Aryl Hydrocarbon Receptor (AhR)-Linked Mitochondrial Program Driver

KYN is not a passive precursor [50]. It can function as an AhR ligand in its own right, shifting transcriptional programs that regulate BNIP3 and PINK1-linked mitophagy, oxidative metabolism, and ROS buffering [155,172]. This means receptor to mitochondria coupling is not exclusive to KYNA [155,190]. It can also arise from the pathway branch point metabolite that rises during inflammation, cancer, and chronic stress states [50,223,224].

### 2.7. 3-Hydroxykynurenine (3-HK), Redox Cycling, and Mitochondrial Injury Endpoints

3-HK behaves like a spark in dry grass [15]. In some contexts, it amplifies oxidative chemistry through autoxidation and glutathione depletion; then, the mitochondrion pays the bill via impaired TCA flux, ROS escalation, and apoptosis [15,51]. That redox pressure can also reprogram immune signaling, so 3-HK links mitochondrial damage to immunometabolic state rather than merely reporting it [47,225].

### 2.8. Quinolinic Acid (QA), N-Methyl-D-Aspartate (NMDA) Signaling, and QA to Nicotinamide Adenine Dinucleotide (NAD^+^) Plus Constraints

QA sits at a double junction [14,94]. It is a precursor for de novo NAD^+^ synthesis, yet it can also drive excitotoxic and oxidative stress through NMDA-linked Ca^2+^ loading and downstream mitochondrial dysfunction when it accumulates or when conversion capacity is limited [14,198,206]. The mechanistic question in Section 2 is whether QA is being cleared into NAD plus or accumulating as a mitochondrial stressor, while Section 3 details how this branch choice rewires NAD plus pools, TCA throughput, and redox ratios at scale [14,94].

**Table 2:** Receptor targets of KYNA and metabolite-driven mitochondrial endpoints of KYNs.

Target	Mitochondrial Effects	Animal Model/Cell Type	Key Findings	References
GPR35	-ATP preservation -ATP turnover ↑ -Mitochondrial oxidative capacity ↑ -ROS production ↓ -mPTP opening inhibition -Calpain-1/2 activity ↓	Myocardial ischemia/reperfusion (rat, NMVMs) Myocardial ischemia/reperfusion (mouse, heart tissue) C57BL/6J mice, adipose tissue	-Receptor activation stabilizes ATP synthase, maintains ΔΨm, prevents ATP hydrolysis, limits ROS and apoptosis -GPR35 blockade reduces calpain-mediated proteolysis, preserves mitochondrial integrity, and mitigates oxidative stress -KYNA enhances PGC-1α expression, promotes mitochondrial biogenesis, increases O_2_ consumption, and initiates anti-inflammatory cytokine production	[16,22,110,126,139,218]
mt GPR35	-ATP synthase dimerization -ATP preservation via the inhibition of ATP hydrolysis	GPR35 knockout mice and neonatal cardiomyocytes	-Binds to ATPIF1 and associates with the mitochondrial outer membrane -Inhibits mitochondrial adenylate cyclase and thereby PKA -Allows ATPIF1 to promote ATP synthase dimerization and prevent ATP hydrolysis	[22,139]
AhR	-Mitophagy (BNIP/PINK1–Parkin) ↑ -ROS production ↓ -ATP preservation -Oxidative metabolism ↑	Hepatocytes, AML12 cells, IPEC-J2 cells, AhR knockout mice, primary hepatocytes	-KYNA and KYN activate AhR to induce PINK1 and BNIP3 expression, promoting mitophagy and preserving mitochondrial respiration under stress -Loss of AhR impairs mitochondrial quality control and increases ROS accumulation, disrupting energy metabolism	[155,172,184,226,227]
mt AhR	-ATP synthase regulation -Fine-tuning ROS production	Mitochondrial fraction, liver cells	-Mitochondrial AhR interacts with ATP5α1; its localization and activity depend on ligand status, possibly influencing ATP synthesis and redox balance	[226]
NMDA-R	-Ca^2+^ influx ↓ -mPTP opening ↓ -Cytochrome c release ↓ -Bcl-XL expression ↑ -Apoptosis ↓ -Complex I coupling	Neurons, microglia, neuronal cultures	-KYNA blocks NMDA-R at the glycine site, limits Ca^2+^ overload, prevents mPTP opening, and protects against apoptosis -Potential crosstalk with complex I regulates bioenergetics	[196]
mt NMDA-R	-Ca^2+^ flux modulation -Fine-tuning ROS production	Rat heart mitochondria	-NR1/NR2B subunits detected in mitochondria -Regulation of ROS production and Ca^2+^ level under hypoxia/ischemia	[197]
α7nAChR	-Regulates Ca^2+^ flux, ROS production, and cytochrome c release via interaction with VDAC1 -Influences mPTP opening and OXPHOS activity through kinase signaling (PI3K/Akt, CaM, Src). -Limits apoptosis through the regulation of Bcl-2/Bcl-xL and caspases	Isolated mouse liver mitochondria U373 human glioblastoma astrocytoma cells KAT II knockout (KAT II^−^/^−^) mice	-α7nAChR–VDAC1 and α7nAChR–Bax complexes identified; receptor inhibition (methyllycaconitine) suppresses cytochrome c release; stimulation (PNU 282987) enhances it; acetylcholine reduces ROS -Decreased KYNA levels increase α7nAChR activity; α7nAChR activation linked to neuroprotection and anti-apoptotic signaling; KYNA may physiologically regulate the receptor	[37,213,215,228,229]
mitoKATP channels (?)	-Channel opening reduces mitochondrial Ca^2+^ overload, delays mPTP opening, and supports cellular survival during ischemic or oxidative stress; -Its function is modulated by GPR35 and ROS signaling	In vivo ischemia–reperfusion models: cardiac and neuronal mitochondria	-Diazoxide (mitoKATP opener) confers neuro- and cardioprotection; inhibition (5-hydroxydecanoate) abolishes protective effects -ROS and GPR35 signaling may crosstalk to regulate mitoKATP and mPTP	[216,217,218]
Complex I/Complex III redox sites (?)	-Redox-sensitive cysteine residues regulate ROS generation (superoxide, H_2_O_2_); transient ROS acts as signaling for stress adaptation -KYNA may scavenge radicals and preserves electron transport	Isolated mitochondria; *C. elegans*	-KYNA may reduce ROS at Complex I and III independent of receptor mechanisms; mild Complex I ROS prolongs lifespan in *C. elegans*; antioxidant effects support mitochondrial stability.	[219,220,221,222]

Abbreviations: GPR35, G protein-coupled receptor 35; ATPIF1, ATP synthase inhibitory factor 1; KYN, kynurenine; KYNA, kynurenic acid; PKA, protein kinase A; ΔΨm, mitochondrial membrane potential; ROS, reactive oxygen species; mPTP, mitochondrial permeability transition pore; PGC-1α, peroxisome proliferator–activated receptor gamma coactivator 1-alpha; AhR, aryl hydrocarbon receptor; mt AhR, mitochondrial aryl hydrocarbon receptor; BNIP3, Bcl-2/adenovirus E1B 19 kDa-interacting protein 3; PINK1, PTEN-induced kinase 1; NMDA-R, N-methyl-D-aspartate receptor; mt NMDA-R, mitochondrial N-methyl-D-aspartate receptor; Bcl-XL, B-cell lymphoma extra-large (anti-apoptotic protein); VDAC1, voltage-dependent anion channel 1; α7nAChR, α7 nicotinic acetylcholine receptor; mitoKATP (mKATP), mitochondrial ATP-sensitive potassium channel; OXPHOS, oxidative phosphorylation.

## 3. Crosstalk Between the KYN Metabolism and the TCA Cycle

Section 3 addresses Objective 2 by focusing on shared metabolic control points. We connect QA-fueled NAD^+^ biogenesis to TCA wiring, redox ratios, and respiratory throughput, then use these nodes to explain how shifts in KYN pathway flux become mitochondrial phenotypes in immune and neural contexts.

### 3.1. Shared Metabolic Intermediates and Redox Balance

Shared intermediates choreograph redox balance across compartments. TCA cycle flux supplies reducing power and precursors, while anaplerosis preserves pool sizes that sustain NADPH and glutathione buffering [3,8]. When flux falters, ATF4 programs rewire amino acid and antioxidant metabolism [1]. Malic enzyme-1 senses malate to pyruvate and tunes NADPH output [11]. Meanwhile, ROS encode signals that reshape metabolic set points and defenses [18,19,20].

Mitochondrial respiration reads the NAD^+^/NADH ratio as a control signal that tunes flux through the TCA cycle, the electron transport chain, and fate decisions in diverse lineages [1,5]. Pool size and localization matter. Mitochondrial carrier transporting NAD^+^ (MCART1) imports NAD^+^ to sustain matrix reactions, while deficits collapse dehydrogenase activity and oxygen consumption [4,6]. Oxaloacetate generated by malate dehydrogenase 2 (MDH2) selectively restrains complex II and reroutes electron flow, thereby reshaping the redox couple in real time [10]. Nicotinamide nucleotide transhydrogenase coordinates nicotinamide adenine dinucleotide (NADH) with nicotinamide adenine dinucleotide phosphate (NADPH) demand, stabilizing redox poise during shifts in glutamine and glucose use [15]. When respiration stalls, serine-driven NADH accumulates and throttles biosynthesis [11].

Immune and disease contexts expose the same logic. LKB1-dependent mitochondrial programs and thioredoxin circuits sculpt NADH turnover, with consequences for chromatin state and T cell effector function [2,13]. Cells with succinate dehydrogenase (SDH) lesions adopt alternative aspartate synthesis routes that hinge on matrix NAD^+^/NADH, salvaging growth despite impaired cycling [7]. De novo NAD^+^ from the KYN metabolism supports macrophage respiration and systemic redox communication, linking Trp catabolism to respiratory control across tissues [3,12,18]. ROS then operate as graded messengers downstream of the ratio, reinforcing or reprogramming signaling pathways [19,20]. Therapeutically, targeted manipulation of NAD^+^/NADH can complement defective electron transport and may slow age-related decline [8,16].

QA sits at the fulcrum of de novo NAD^+^ synthesis, converting Trp catabolism into respiratory capacity. In macrophages, intact quinolinate to NAD^+^ flux sustains complex I-driven oxidation and immune effector programs; aging and inflammation magnify the dependence, and pathway blockade collapses oxygen consumption [1]. In tissues under ischemia reperfusion, diversion away from quinolinate depletes NAD^+^, weakens antioxidant defenses, and heightens oxidative injury, all reversible with NAD^+^ augmentation [3]. Genetic and clinical data converge on QPRT as a bottleneck whose repression or loss yields quinolinate accumulation, reduced NAD^+^, and vulnerability to damage [9,13]. Astrocytes and neurons similarly require quinolinate conversion to maintain SIRT activity, viability, and mitochondrial function during neuroinflammation [8].

Evolution supplies redundancy and reach. Yeast can secrete and reimport quinolinate to stabilize NAD^+^ pools, and, when canonical steps fail, UMPS can substitute to complete synthesis [4,11]. Engineered circuits that route quinolinate toward NAD^+^ raise cellular NAD(H) and bolster electron transport, illustrating design principles for metabolic support [5]. Across physiology and disease, rising mitochondrial work rates are associated with higher circulating quinolinate and nicotinamide, consistent with coupled biogenesis and electron transport chain (ETC) demand [10]. Yet excess or chronic pathway activation is harmful, impairing bioenergetics in neurons and reshaping tumor growth constraints, where ubiquinol oxidation remains a nonnegotiable requirement beyond NAD^+^ regeneration alone [12,17]. Together, these findings position QA as both a sensor and supplier that links Trp flux to NAD^+^ homeostasis, redox poise, and sustained respiratory throughput [2,6,7,14,15,16,18,19].

α-Ketoglutarate sits at the crossroads of carbon flow and immune control, shaping how cells route Trp into the KYN metabolic pathway. Through IDH2-dependent reductive carboxylation, α-ketoglutarate fuels isocitrate and citrate production, generates NADPH, and thereby tunes redox poise that can favor or restrain IDOs and TDO activity through cofactor availability and metabolic context [1,2]. Inflammatory signaling rewires this hub. Type I interferon inhibits isocitrate dehydrogenase, distorting the citrate to α-ketoglutarate ratio, shifting mitochondrial electron supply, and altering the local redox environment that licenses Trp catabolic flux [3].

Macrophage polarization provides a vivid example. Network integration reveals a metabolic break at IDH in M1 cells, fragmenting the TCA cycle and lowering α-ketoglutarate regeneration; the result is constrained anaplerosis, altered NADPH production, and a redox profile conducive to heightened immune effector programs that intersect with IDO induction [5]. In tumors, nutrient competition and hypoxia reshape the same nodes. The microenvironment modulates α-ketoglutarate levels and the balance between oxidative and reductive TCA routing, which in turn conditions IDO1 and TDO2 expression and the effectiveness of their pharmacologic blockade [4]. Across aging and chronic inflammation, these α-ketoglutarate-centered adjustments integrate energy metabolism with Trp fate, coupling respiratory control to immunoregulatory enzyme flux [2] (Table 3).

### 3.2. Immunometabolic Integration

Immune activation tilts Trp fate toward the KYN axis, lowering substrate and generating metabolites that reprogram effector circuits [10,15,19]. KYN and allied ligands engage AhR, dampen T cell proliferation, and favor regulatory programs, shaping tolerance in infection, autoimmunity, and cancer [1,3,4,11,18]. Context matters. Inflammaging sustains this reflex, while serotonin–KYN balance and metabolic feedback refine outcomes [12,14,20]. KYN to Trp ratios index pathway load, and Rab4A-dependent mTOR signaling links mitochondrial metabolism to KYN sensitivity [5,13].

Succinate links carbon flux to inflammatory licensing. In lipopolysaccharide-challenged macrophages, accumulation of succinate stabilizes hypoxia-inducible factor 1 alpha (HIF-1α), boosts glycolysis, and elevates IL-1β transcription and release [2,10]. Mitochondrial control is pivotal. Inhibition or retuning of SDH rewires electron flow, sustains HIF-1α, and aggravates tissue injury, while SUCNR1 signaling extends succinate’s reach to endothelium and epithelium, amplifying cytokines and vascular pathology [1,6,9,13,15]. Parallel cues converge. STING activation raises succinate and locks HIF-1α-dependent effector programs during infection; L-2-hydroxyglutarate similarly enforces the HIF-IL-1β axis [3,11,14].

Context shapes outcome. Pro-inflammatory cytokines further potentiate HIF-1α, embedding a feedforward loop, yet SDH also supports STAT3-driven IL-10, revealing countervailing anti-inflammatory circuitry within the same module [12,13,17]. Upstream metabolic governors, including SIRT6, adjust succinate levels and HIF-linked glycolysis in pathogen-challenged macrophages [18]. In vascular and synovial beds, pharmacologic or nutraceutical inhibitors that blunt succinate accumulation or HIF-1α activation reduce IL-1β output, neovascularization, and plaque inflammation, illustrating therapeutic tractability across diseases characterized by immunometabolic stress [4,16,19,20,28,203,254]. Together, these data position the succinate–HIF-1α axis as a tunable rheostat that integrates mitochondrial respiration with inflammatory effector commitment.

#-HK sits at a volatile intersection of metabolism and immunity, where its redox cycling can both ignite and quench oxidative chemistry. Autoxidation and dimerization generate ROS that deplete glutathione, derail the TCA cycle, and trigger apoptosis, yet context permits radical scavenging and neuroprotective outcomes [3,4,5,6,7]. In vascular, renal, and neuroinflammatory states, elevated 3-HK tracks with oxidative stress and endothelial dysfunction, positioning this metabolite as a sentinel of immunometabolic strain [1,5,6].

These redox swings feed signaling loops that tune inflammatory tone. ROS linked to 3-HK amplify IL-6 and IL-1-driven programs, prime monocytes through mTOR-dependent circuits, and shape T cell activation thresholds and lineage decisions [2,8,9,10,11,12]. Feedback control is nuanced. Mitochondrial injury can proceed with or without early ROS surges, implying parallel mitochondria-centered sensors and effector arms downstream of 3-HK [13]. Collectively, 3-HK orchestrates bidirectional crosstalk between metabolism and immunity, coupling Trp flux to ROS governed transcriptional and epigenetic checkpoints that determine tolerance versus tissue injury [1,4,7,10,11,12].

At the interface of Trp catabolism and the TCA cycle, a set of metabolic checkpoints governs whether immunity accelerates or brakes. Enzymes such as IDO1, TDO, and KMO gate substrate flow to KYN and downstream ligands that converge on AhR, adjusting effector programs, tolerance, and chronic inflammation [1,2,3]. This control integrates with organelle level routing of carbon and reducing equivalents, where cytosol–mitochondria handoffs shape glycolysis–TCA coupling and enforce redox thresholds for immune activation [8,9,11]. De novo NAD^+^ synthesis from KYN intermediates adds another lever, feeding respiratory capacity and feedback to transcriptional fate decisions in aging and cancer [6,12].

Checkpoint behavior is contextual. In T cells, Rab4A-controlled endosomal traffic intersects with KYN-sensitive mTOR signaling to couple mitophagy, nutrient transport, and lineage specification [4]. Tumors exploit depletion and signaling in parallel, creating an immunosuppressive niche that often resists single-agent IDOs or TDO blockade, arguing for combinations that co-target metabolic nodes and canonical immune checkpoints [2,5,13,15,18]. Microbial Trp products reinforce AhR-dependent suppression in tumor-associated macrophages, linking diet and microbiota to checkpoint tone [16]. Stress programs that arise when TCA flux is curtailed activate ATF4 and remodel amino acid and redox metabolism, thereby re-indexing sensitivity to Trp pathway control [14]. Multi-omic maps now resolve these modules in human macrophages and guide rational intervention across inflammatory disease and oncology [10,17,19].

### 3.3. Pathological Implications

From circuits to clinics, dysregulated Trp–KYN and TCA nodes shape disease trajectories across brain, vasculature, metabolism, and cancer. Imbalanced metabolites drive neurotoxicity, immune exhaustion, and cardiometabolic risk, while altered NAD^+^ supply and ATF4 programs expose vulnerabilities [3,4,5,6,8,9,10,12,13,14,15,16,17,18]. Translational paths now pair pathway modulation with tumor bioenergetic targets and immune recalibration [6,12,13,14,16].

Excess QA and diminished KYNA form a pathogenic redox and excitotoxic dyad in neurodegeneration. QA engages NMDA-Rs, drives mitochondrial ROS, and depresses respiratory capacity, creating a feedforward loop of bioenergetic failure and inflammation [2,5,16]. By contrast, KYNA buffers glutamatergic stress and restores antioxidant defenses, including Nrf2 signaling, yet is frequently reduced in Alzheimer’s and Parkinson’s disease [3,5,6,19]. Clinical and biomarker studies converge on elevated QA to KYNA ratios across aging and disease, linking this imbalance to tau and amyloid burden, neuronal dysfunction, and faster progression [49,53,255]. Therapeutically, redirecting flux away from QA and enhancing KYNA, for example with KMO inhibition or pathway modulation, offers a rational strategy to stabilize mitochondria and slow neurodegeneration [4,18,20].

Psychiatric disorders display a characteristic coupling of immune tone and bioenergetics, centered on a rerouting of Trp toward KYN metabolism [49,256,257]. Meta analyses and cohort studies reveal reduced Trp and KYN with imbalanced neurotoxic and neuroprotective metabolites, tracking mood, psychosis, and cognitive deficits [2,3,4,6]. Immune activation accelerates this shift, depresses serotonin, and favors QA, with state-dependent oscillations across acute and remitted phases [10,11,12,18,19]. These molecular changes map onto mitochondrial and synaptic function, and show moderate blood–brain concordance for select metabolites that can index symptom burden and progression [13,16]. Mechanistically, KYN reprogramming may be compensatory or pathogenic, often both; therapeutic strategies now target enzymes and flux control while integrating microbiome sensitive modulators and trial readouts [1,5,7,14,15,258].

Tumors co-opt Trp catabolism to choreograph immune escape while fueling growth. IDO1 and TDO2 divert substrate toward KYN, which activates AhR, expands Tregs, and exhausts cytotoxic T cells; clinical experience shows that single-enzyme blockade often falters, underscoring redundant wiring and the need for multi-target strategies [1,2,3,4,6,12,15]. Beyond initiation, downstream nodes such as KMO and kynureninase (KYNU) shape metastatic behavior, stromal crosstalk, and chemoresistance, particularly in aggressive breast and renal cancers [5,7,8,14,16].

**Figure 3:**
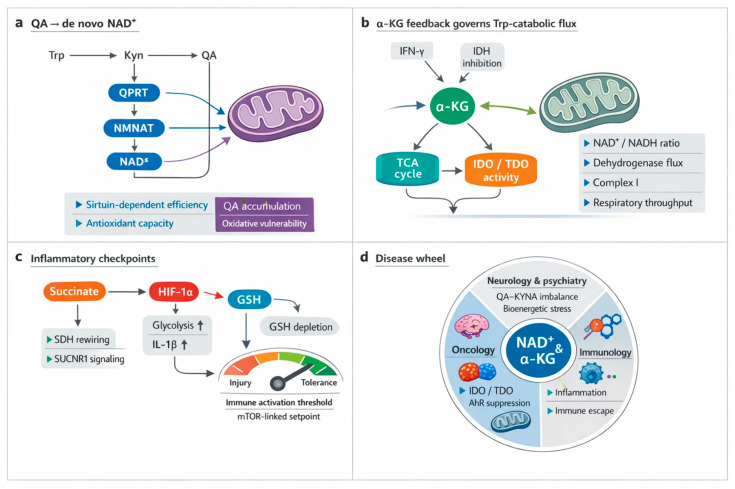
Bridging KYN metabolism and the TCA cycle via NAD^+^ biogenesis, alpha KG feedback, and inflammatory checkpoints. (**a**) QA feeds the QPRT NMNAT NADS axis to expand NAD(H) pools and tune NAD^+^/NADH, dehydrogenase flux, CI-driven oxidation, and respiratory throughput; impaired QA to NAD^+^ conversion promotes QA accumulation, NAD^+^ depletion, and oxidative vulnerability during inflammation or ischemia. (**b**) α-KG acts as a flux governor linking carbon routing and redox context to IDO TDO rate setting and KYN output, with immune cues, IDH inhibition, and TME constraints reshaping alpha KG regeneration and NADPH buffering. (**c**) Succinate HIF 1 alpha and 3 HK redox cycling amplify glycolysis, IL 1 beta, SDH and SUCNR1-driven inflammatory tone, and ROS glutathione stress that shifts mTOR-linked activation thresholds. (**d**) Disease wheel maps these nodes onto neuropsychiatry and oncology via IDO TDO AhR rewiring and QA KYNA imbalance. 3-HK, 3-hydroxykynurenine; AhR, aryl hydrocarbon receptor; CI, Complex I; HIF-1α, hypoxia-inducible factor 1 alpha; IDH, isocitrate dehydrogenase; IDO, indoleamine 2,3-dioxygenase; IL-1β, interleukin 1 beta; KYN, kynurenine; KYNA, kynurenic acid; mTOR, mechanistic target of rapamycin; NAD(H), nicotinamide adenine dinucleotide (oxidized and reduced forms); NAD^+^, nicotinamide adenine dinucleotide; NADS, NAD synthetase; NMNAT, nicotinamide mononucleotide adenylyltransferase; QA, quinolinic acid; QPRT, quinolinate phosphoribosyltransferase; ROS, reactive oxygen species; SDH, succinate dehydrogenase; SUCNR1, succinate receptor 1; TCA, tricarboxylic acid cycle; TDO, tryptophan 2,3-dioxygenase; TME, tumor microenvironment; alpha KG, alpha-ketoglutarate.

Therapeutic concepts increasingly pair immune checkpoint inhibitors with metabolic rewiring. KYNU depots deplete intratumoral KYN and synergize with programmed cell death protein 1 (PD-1) blockade, while small molecules like icariside I attenuate AhR signaling and restore effector function [9,10]. Remodeling the glutathathione peroxidase 4 (GPX4)–KYNU axis or broader tumor microenvironment (TME) metabolism reprograms macrophage polarization and dismantles suppressive niches, offering tractable paths to overcome resistance [11,13,18,19,20] (Table 4, Figure 3).

## 4. Analytical Strategies for Simultaneous Quantification

The combined measurement of the TCA and KYN metabolic pathway in a single method has attracted significant interest in the fields of neurology, oncology, and mitochondrial biology [13,14,260]. While the two metabolic pathways are typically studied separately, combined studies are increasingly sought after [65,261,262,263]. This allows for the investigation of interactions between pathways underlying disease phenotypes in a single method, without separate preparations, and by eliminating inter-assay variance. However, designing a single analytical method requires consideration of chromatographic incompatibilities, ionization, and polarity differences [264,265,266]. In addition, several orders of magnitude variations in biological samples for some metabolites must be taken into account, which weakens the usefulness of an already well-developed analytical method [267,268,269]. This section explains the fundamental challenges of the simultaneous measurement and presents practical engineering solutions. Finally, we would like to highlight the validation standards required in clinical research, similar to diagnostic applications, where the methods used must meet serious criteria, for which appropriate guidelines are available [270,271].

### 4.1. Rationale for Unified Measurement

The scientific and clinical promise of simultaneous quantification lies in minimizing fragmentation in experimental workflows [272,273,274]. Instead of separate assays for TCA intermediates and KYNs, a unified measurement allows for a simultaneous overview of cellular energetic processes and neuroactive metabolism [275,276]. Common measurement improves efficiency, reduces sample burden, and makes meaningful comparisons between groups more effective [277,278,279].

Metabolomic studies often struggle with limited availability. In cerebrospinal fluid (CSF), where lumbar punctures yield only a few hundred microliters, or in animal studies, where only a few microliters are available, distributing aliquots to multiple specialized assays reduces the measurement options, thereby compromising the results or the statistical power of the work [280,281,282]. Similarly, plasma samples collected in efficacy studies may be limited by ethical and logistical considerations, while tissue biopsies are often only available in milligram quantities [283,284,285]. When TCA intermediates and KYN derivatives are measured in separate analytical runs, each uses a portion of the already small amount of biological sample, which reduces measurement efficiency and may lead to the possibility of missing biologically important correlations [286,287,288,289]. Simultaneous measurements in single-run assays eliminate the need to split the sample volume into multiple aliquots, and metabolite relationships within it can be examined more reliably [268,287,290,291]. These measurements also allow the application of multivariate statistical models that integrate data collected from mitochondrial and KYN metabolites and increase the potential for cross-metabolic pathway analysis, allowing for more reliable quantification of ratios such as succinate/quinolinate ratios or citrate-KYNA correlations in psychiatric and neurological diseases [13,14,234,245,292,293,294,295,296,297].

A major obstacle to reproducibility in biomarker research is inter-assay variability. Separate LC-MS methods, independently optimized for TCA intermediates and KYNs, differ in chromatographic stationary phases, gradient conditions, derivatization techniques, and ionization polarity [96,263,298,299]. Harmonized measurement reduces technical heterogeneity by incorporating both metabolic pathways into a single analytical measurement, ensuring that co-eluting matrix components and ion suppression phenomena are consistent across analytes [268,299,300,301]. This is particularly important for meta-analyses and longitudinal cohort integration, where cumulative error from multiple platforms can mask biologically significant differences [97,302,303]. Unified workflows also simplify QC procedures and validations, as pooled reference samples, system proficiency tests, and internal standards can be used globally [270,304,305,306]. This ultimately results in improved reproducibility, increased statistical reliability, and comparability between stronger studies—key requirements for moving beyond exploratory research to regulatory-level biomarker validation [307,308].

In addition to consistency within a single assay, standardized analytical workflows also facilitate inter-center comparability, allowing for standardization and the establishment of international cutoffs instead of results with diverse and high variance [73,309]. They are also characterized by long-term reproducibility and reliability [310,311,312]. When laboratories use identical chromatographic conditions, calibration strategies, and internal standard placements, the correction of batch-to-batch drift and instrument-specific biases becomes significantly more reliable, although in practice this is almost impossible due to the large variety of chromatographic systems, columns, and mass spectrometers available [313,314,315,316].

### 4.2. Chromatographic and Mass Spectrometric Challenges

Simultaneous detection of TCA intermediates and KYNs can be analytically challenging, if not impossible [263,288,317,318]. TCA acids are highly polar, have low molecular weight, break easily at low voltages, are poorly retained in reversed-phase systems, and are often measured in negative electrospray mode [263,319]. In contrast, KYNs exhibit different polarity, aromaticity, and proton affinity, and are typically measured in positive electrospray ionization (ESI) mode [68,288,320,321,322]. Reconciliation of these different physicochemical properties defines the fundamental challenge of chromatography and ionization [321,323,324,325].

TCA intermediates, such as citrate, α-ketoglutarate, and malate, are highly polar, low-molecular-weight organic acids that elute early on conventional C18 columns and exhibit poor retention [289,326,327]. Ion-pairing agents or hydrophilic interaction chromatography (HILIC) are often required for proper separation [328,329,330,331]. However, metabolites of KYN metabolism range from more neutral, aromatic amino acid derivatives (KYN, KYNA) to highly acidic, low-molecular-weight, simple molecular structures with few transitions (QA, picolinic acid), requiring a broader range of chromatographic selectivity [298,332,333,334]. Choosing a mobile phase that is capable of retaining both classes of molecules requires a balance between retention and ionization efficiency [265,335]. Formic acid is a common additive used for positive ion detection, while ammonium acetate or ammonium formate buffers at neutral-basic pH help negative-mode detection by enhancing ionization [265,336,337]. Buffers with high ionic strength can suppress the electrospray response, especially for aromatic KYNs [321,338,339]. Consequently, hybrid strategies—slightly buffered aqueous phases with carefully screened organic modifiers—are used to retain acids without unduly compromising positive-mode sensitivity [265,340,341,342]. Such trade-offs reflect the trade-off between chromatographic retention and MS compatibility [265,340,343].

The polarity of electrospray ionization fundamentally affects the quality of the signal, and some molecules only give signals in one polarity mode [321,344,345,346]. TCA cycle intermediates ionize best in the negative mode, forming stable deprotonated species [263,289,347]. KYNs, especially KYN and KYNA, favor the positive mode through protonation or ammonium adduct formation [68,248,348]. Trying to quantify both in a single run either forces a polarity switch within a gradient or the acceptance of one polarity, which can cause a decrease in sensitivity of up to several orders of magnitude for some analytes [266,349,350]. The polarity switch causes residence time losses when the analytes elute closely [266,351,352]. Furthermore, the ion source parameters—desolvation temperature, gas, and capillary voltage—should be set differently for acids and aromatics [353,354,355,356]. Adduct formation further complicates the process: sodium and potassium adducts are common for citrate and succinate, while ammonium adducts affect KYNs [357,358,359]. Without careful control, the diversity of ionic species reduces quantitative reproducibility [360,361,362,363]. Thus, the challenge is not only chromatographic but also electrochemical, requiring source-level design or the use of stable isotope-labeled internal standards to normalize for adduct heterogeneity [364,365,366,367].

Another critical difficulty arises from concentration differences. TCA intermediates such as lactate, pyruvate, citrate, malate and succinate often circulate in the plasma in the high micromolar and millimolar range, while downstream KYNs such as 3-hydroxykynurenine, xanthurenic acid, anthranilic acid, QA or picolinic acid are present in the nanomolar range [249,275,368,369,370]. This difference spans four to six orders of magnitude, exceeding the linear dynamic range of almost all detectors used in mass spectrometers [340,371,372]. Injecting samples to achieve adequate sensitivity for low amounts of KYNs carries the risk of saturating the signals of high amounts of TCA metabolites, leading to peak distortion and poor quantitation, while diluting samples to TCA concentrations below the detection limit of kynurenes can reduce detection limits [340,373,374]. Signal compression and ion suppression further exacerbate the problem when high concentrations of acids dominate the spray cloud [375,376,377,378]. These concentration dynamics require either double injections (concentrated and many-fold diluted) or special detector linearization approaches that carefully span the entire range [379,380,381]. Without such adaptations, single-run assays run the risk of erroneously measuring either low-abundance KYN metabolites or concentrated TCA intermediates [240,382].

### 4.3. Practical Solutions

Co-measurements with physicochemical and quantitative differences between TCA and KYN metabolites require considerable foresight and planning [68,383]. To solve this problem, advanced chromatographic approaches such as HILIC, mixed mode or multidimensional separations are available, but advanced use of the mass spectrometer is also essential, such as polarity switching and properly optimized ion source conditions [266,383,384,385]. The guiding principle is comprehensive metabolite coverage without sacrificing sensitivity or reproducibility.

Strong retention of polar acids provides better separation of TCA intermediates, while less polar KYNs can be adequately retained by selecting the appropriate eluent composition, gradients, column temperature, eluent composition, and pH. Mixed-mode columns, which combine ion-exchange and reversed-phase characteristics, offer another option, improving resolution across different chemical classes (e.g., ZIC-cHILIC, BEH Amide, BEH HILIC HILIC Silica, or Accucore HILIC) [68,278,386,387]. Even more precise, but more complex, are two-dimensional LC (2D-LC) systems, where an initial HILIC or ion-exchanger binds the highly polar acids, followed by reversed-phase separation of the aromatic kynurenes [388,389]. Although technically demanding, 2D-LC allows the analysis of both metabolite classes in a single workflow [390,391,392]. They also have the disadvantage of being complex, extremely error-prone and difficult to implement, especially in biological matrices [390,393]. A high degree of chromatographic and engineering expertise is required to design and operate such a complex system efficiently [394,395,396]. The piping, switching valves, and dead volume must be optimized to preserve peak shape and sensitivity [397]. Nevertheless, these approaches illustrate how chromatographic ingenuity can reduce polarity bias and expand assay coverage [328,398,399].

A practical compromise is to design two complementary LC-MS runs instead of forcing all analytes into a single chromatographic measurement [269,400,401]. For example, an optimized and validated run in negative mode for TCA intermediates and a second run in positive mode for KYNs allows for adequate sensitivity in both cases [73,263,402,403]. Although this doubles the run time, it can solve the problem of multiple sample preparation and sample volumes, and it also maintains quantitative reliability, but in return avoids retention or ionization compromises [286,393,402,404]. Hybrid approaches also exist: a single-run method that uses precisely timed polarity reversals at appropriate times in the gradient can measure both acidic and aromatic metabolites with acceptable reproducibility [264,405,406,407,408]. Advances in high-speed electronics optimization have reduced the time-dependent degradation of polarity reversal measurements, making single-run hybrids increasingly feasible [266,349,400]. The choice between two-run and single-run strategies depends on the scale of the study, throughput requirements, and instrumentation, with hybrid methods being preferred for exploratory studies and two-run strategies for large clinical cohorts where reproducibility overrides throughput [268,269,409,410,411].

**Figure 4:**
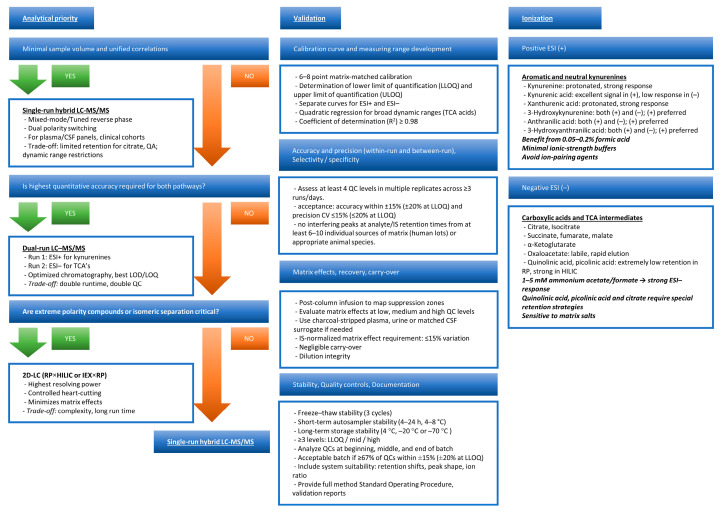
Decision framework and validation pathways for LC–MS/MS quantification of KYN pathway metabolites and TCA intermediates. Practical decision framework and validation roadmap for targeted LC–MS/MS quantification of KYN pathway metabolites and TCA intermediates. Method choice is guided by sample volume, polarity range, quantitative rigor, and chromatographic feasibility. Single-run hybrid LC–MS/MS suits limited-volume clinical matrices but may compromise retention of highly polar acids. Dual-run strategies (ESI^+^ for KYNs; ESI^−^ for TCA acids) maximize sensitivity and chromatographic performance at the cost of run time and QC burden. For extreme polarity or isomeric overlap, 2D-LC provides orthogonal separation with added complexity. Validation includes matrix-matched calibration, defined LLOQ/ULOQ, accuracy and precision across ≥4 QC levels, and assessment of interferences, stability, recovery, carryover, and matrix effects. Ionization mode selection is critical, with challenging analytes requiring tailored retention and suppression control. 2D-LC, two-dimensional liquid chromatography; ESI^+^, positive electrospray ionization; ESI^−^, negative electrospray ionization; KYN, kynurenine; LC–MS/MS, liquid chromatography–tandem mass spectrometry; LLOQ, lower limit of quantification; QC, quality control; TCA, tricarboxylic acid (cycle); ULOQ, upper limit of quantification.

Internal standards (IS) labeled with stable isotopes (D, C^13^, N^15^) are essential for reliable and reproducible mass spectrometry measurements [365,412,413]. They correct for matrix effects, ion suppression and adduct variability in both metabolite classes [412,414,415]. The choice of IS should be carefully considered due to their extremely high cost and availability [304,416]. Optimally, each metabolite to be measured would have its own stable isotope-labeled IS, but this would significantly increase the cost [413,417,418]. It is worth considering what the goal is, which metabolites can be combined under a common IS, and incorporating them at the earliest possible step, ideally during sample extraction, which compensates for losses during preparation and ensures normalization between runs [419,420,421,422]. For studies spanning a wide concentration range, multiple concentration-matched calibration curves recorded by IS prevent saturation in the high range while maintaining sensitivity for trace analytes [423,424,425]. Without this strategic use of isotopes, uniform workflows risk systematic bias in both chemical classes [420,426,427] (Figure 4).

### 4.4. Validation and Clinical Feasibility

Rigorous validation is required for a simultaneous TCA-KYN metabolite assay to become a clinical or research method [263,287,400,428]. Guidelines governing mass spectrometry methods require reproducibility testing, both inter- and intra-day, linearity, limit of detection, lower limit of quantification (LLQ) determination, carry-over, precision and accuracy, matrix effect, recovery, selectivity and stability testing [429,430,431].

An important step in validation is the linearity of the calibration curve, the determination of dynamic ranges, the measurement of matrix effects, and the stability of the samples [432,433,434]. The linearity range should be excellent (R^2^ > 0.98) from the nanomolar range to the millimolar range depending on the metabolite [400,435,436,437]. To calculate the matrix effect, a standard solution or different matrices need to be spiked for each matrix to be measured [438,439,440,441]. A mandatory element of stability studies is the performance of multiple freeze–thaw tests, and it is also important to test the stability of samples under different conditions (e.g., room temperature, 4 °C, −20 °C, and −80 °C) [442,443,444,445,446]. Reproducibility is used to determine the differences between tests performed on the same day and on different days, which is an important basis for long-term QC [447,448,449,450]. Validations are accompanied by rigorous documentation, where changing any of the steps can cause critical differences in the results, so a validated system should not be modified without compelling reason, or only with caution and revalidation [451,452].

Several studies have shown that HILIC methods can be suitable for the analysis of both TCA and KYN metabolites with appropriate sensitivity and selectivity [398,407,453,454]. Methods using anion exchange stationary phase exploit the almost unlimited potential of buffers, which can be properly applied by a well-trained analyst [455,456,457,458,459]. Hybrid HILIC-AEX systems are promising for both clinical and research purposes, which can be used with high-resolution MS systems [278,460]. Although suitable assay kits are not yet available on the market for a wide range of metabolites, especially for TCA and PK, their appearance in the future should be expected due to the high demand [304,461,462,463]. With the continuous optimization of diverse stationary phases, polarity switching protocols and isotope calibration, combined assays may soon provide robust, validated tools for integrative mitochondrial and immuno-metabolic diagnostics [264,464,465,466,467] (Table 5).

### 4.5. Potential Clinical Deployment of a Harmonized Tricyclic Acid (TCA)–Kynurenine (KYN) Assay

A coordinated LC-MS-based assay targeting the most important metabolites of the TCA cycle and KYN pathway could be used in a number of clinical studies, such as studies on mitochondrial dysfunction and immunometabolic dysregulation [73,89,287,479]. This section moves from analytical diversity to convergence [73,480]. Rather than exhaustively reviewing all reported LC–MS approaches, we define a minimal, clinically deployable LC–MS/MS strategy for simultaneous quantification of KYNs and selected TCA intermediates, highlighting decision points relevant for routine laboratories [481]. Table 6 is intended as a minimal, trial-ready panel rather than an exhaustive analytical overview [73,480]. Given the differential stability of QA, 3-HK and selected TCA intermediates, harmonized pre-analytical handling (rapid quenching, antioxidant stabilization, and limited freeze–thaw cycles) and the use of isotope-labeled internal standards are essential prerequisites for inter-laboratory comparability. All targeted metabolites can be accommodated using HILIC-based or mixed-mode chromatographic separation, enabling simultaneous coverage of highly polar KYNs and TCA cycle intermediates within a harmonized analytical workflow. KYN pathway metabolites are preferentially detected in positive electrospray ionization (ESI^+^), whereas TCA cycle intermediates exhibit superior sensitivity and robustness in negative ion mode (ESI^−^), supporting polarity-switching or dual-mode acquisition strategies [287,482].

In neuropsychiatric disorders such as major depression, plasma sampling at baseline and post-treatment could be used to monitor Trp, KYNe, KYNA, QA, and selected TCA intermediates, with pharmacodynamic data including KYN/Trp ratios, pathway branching shifts, and markers of systemic metabolism [50,92,206]. It could also be used in acute inflammatory or infectious conditions, including sepsis [486,487,488]. Sampling at multiple time points over 24–48 h can reveal dynamic changes in KYN pathway activation and TCA cycle disruption, reflecting immune activation and metabolic stress [489,490]. In cases of acute or chronic kidney injury, combined plasma and urine testing is essential for accurate assessment [491,492,493,494]. Together, these scenarios illustrate how standardized sampling times and pathway-based readouts allow for the use of a harmonized assay for longitudinal monitoring, assessment of response to treatment, and comparability across studies in different clinical settings [495,496,497,498].

## 5. Discussion: Strengths, Limitations, and Future Perspectives

This section integrates mechanism with measurement. Strong signals emerge where high quality LC–MS workflows align with biology: KYN metabolite panels show diagnostic and prognostic promise, including KYNA in depression and cardiometabolic risk stratification [92,287,499]. Yet uncertainty persists. Static metabolite levels obscure flux and compartmentation, cell lines incompletely mirror tumors, and heterogeneous sampling blurs psychiatric associations [500,501]. Next steps should prioritize standardized protocols and reference materials, longitudinal and tissue-matched cohorts, and fluxomics with stable isotopes to resolve directionality [502,503]. Improved annotation and pathway contextualization, combined with targeted validation and mechanistic interventions, will convert metabolite signatures into clinically actionable tools [504,505].

A final point of positioning matters. Prior syntheses have been strong within their lanes, whether they center on neuropsychiatric signatures, immunoregulatory enzyme control, or NAD^+^-centered metabolic theory. Here, the emphasis is different. We treat mitochondrial readouts as the common currency and then ask which receptor signals and which metabolic branch points most plausibly move those readouts in patients (Figure 1). That framing is why the analytics discussion sits beside receptor biology and de novo NAD^+^ biochemistry rather than being relegated to a technical appendix.

KYNA appears to choreograph mitochondrial resilience through a convergent receptor network [22]. Engagement of GPR35 preserves ATP under stress, tempers Ca^2+^ influx, and restrains NLRP3 signaling, linking membrane cues to organelle protection in ischemia, infection, and metabolic inflammation [22,32]. Parallel signaling through the AhR modulates oxidative programs and inflammatory tone, with context-specific outcomes across brain and cardiovascular systems [190,506]. At excitatory synapses, NMDA-R antagonism by KYNA lowers excitotoxic drive and secondarily shields mitochondrial function [193,507]. α7 nicotinic receptor interactions remain debated, yet may contribute to immune–neuronal crosstalk in select settings [37,214]. Receptor crosstalk is likely, as GPR35 and AhR signals intersect Ca^2+^ handling, bioenergetics, and transcriptional defenses, while gut-derived KYNA can prime GPR35-positive myeloid cells or coordinate epithelial protection via combined AhR–GPR35 sensing [16,508]. Therapeutically, receptor-selective modulation offers leverage to enhance mitochondrial robustness without broad immunosuppression [16,22].

Modern LC–MS has moved the KYN and Trp–NAD^+^ networks from piecemeal panels to near-complete coverage, enabling coordinated profiling across plasma, CSF, urine, peritoneal fluid, tissues, and even subcellular fractions [68,298]. Single-run or low-cost targeted assays quantify core KYN metabolites alongside central carbon nodes, while hybrid targeted–untargeted and solvent-switching workflows expand breadth without sacrificing precision [65,509]. Organelle-resolved strategies using chemical labeling now map compartmental metabolomes, a prerequisite for linking receptor cues to mitochondrial readouts in vivo [510,511]. Comparative cell-line and clinical applications illustrate scalability, drug-screening utility, and disease stratification potential, including acute kidney injury and diabetes [479,512]. Limitations persist. Protocol heterogeneity, matrix effects, and incomplete annotation still blunt cross-study synthesis, motivating harmonized methods, reference materials, and improved pathway enrichment analytics [249,513]. The path forward integrates stable-isotope flux tracing with multi-organ, multi-compartment LC–MS to resolve directionality and translate signatures into actionable endpoints [514,515].

Coupling of the KYN metabolism to the TCA cycle provides a unifying scaffold for NAD^+^ governance and redox control across tissues [14,50]. KYN flux supplies quinolinate for de novo NAD^+^ and tunes mitochondrial function, intersecting with sirtuins, DNA repair, and stress programs [15,94]. Tissue context dictates outcome [14,225]. In liver ischemia–reperfusion, diversion from quinolinate depletes NAD^+^, weakens antioxidant defenses, and heightens injury, whereas augmentation restores resilience [43,516]. Macrophages require KYN-derived NAD^+^ to maintain respiratory capacity and phagocytic competence, especially with aging [14,42]. Neurons and astrocytes similarly depend on KYN metabolite inputs to preserve viability and redox poise [45,230]. Kidney and brain exhibit disease-specific deficits or sex-dependent shifts in KYNs and NAD^+^ pools, linking metabolic imbalance to dysfunction [517,518]. These nodes communicate with NADPH–glutathione circuits that relay mitochondrial workload to the endoplasmic reticulum, completing a cross-organ redox axis [15,519]. Cancer exploits the same wiring to fuel growth and immune escape, emphasizing context-precise intervention [224,244].

Generalization falters when biology and measurement drift by model and tissue. LC–MS workflows vary in extraction, ionization, and matrices, so results hinge on protocol and normalization choices; no single assay spans all key metabolites across compartments [520]. Biological context adds another filter [521]. Enzyme expression, receptor density, and circadian control reshape flux in organ-specific ways, while inflammation rewires the same nodes differently across tissues [522]. Plasma signals often diverge from tumor or CNS readouts, and correlations between serum, CSF, urine, and viscera are imperfect, urging multi-compartment designs with matrix-specific validation [523]. Model choice matters. Disease stage, timing, and targeted enzyme alter outcomes in autoimmune and cancer settings, and cell line metabolism incompletely recapitulates in vivo states [524,525]. Standards are therefore essential. Anchored designs, reference materials, harmonized extraction, and data-driven normalization should precede interpretation, enabling cross-study synthesis and credible biomarker development [313,526].

Progress toward single-run LC–MS coverage of the KYN and Trp–NAD networks is real yet fragmented [66,250]. Panels quantifying 9–11 metabolites in CSF, plasma, urine, or peritoneal fluid prove feasibility, but validation remains matrix specific and inter-laboratory comparability is limited [68,527]. Methods for broader coverage or alternative separations extend analyte space while introducing longer run times, partial validation, or unresolved matrix effects [250,288]. Hybrid targeted–untargeted assays in large cohorts show precision, yet harmonized adoption and cross-site calibration are uncommon [65]. Cell-based workflows accelerate mechanism and screening but are not configured for clinical interchangeability [72,509]. The path forward is methodological convergence: clinical and laboratory standards institute (CLSI) C62-A–aligned development, isotopologue-rich internal standards, commutable multi-matrix calibrators, external quality assessment, and anchored normalization across batches and sites [288,298]. Reporting standards should include matrix-specific recovery, LLOQ, and carryover, plus retention-time locking and shared reference materials, so signatures translate across studies and into routine diagnostics [73,528].

Temporal and spatial resolution remain the weakest links in vivo. Most datasets provide static plasma or CSF snapshots that miss rapid fluxes and compartment shifts documented by disease meta-analyses and multi-compartment comparisons [529,530]. Maternal–fetal gradients and region-specific brain profiles illustrate strong spatial heterogeneity, yet longitudinal and organelle-resolved measurements are rare [531,532]. Cell-type time courses show inducible, phase-specific enzyme and metabolite waves, underscoring dynamics that bulk tissues blur [533,534]. Chronic KMO loss reshapes circulating metabolites without altering whole-body energetics, implying that acute transients, not steady states, drive phenotype [535]. Cross-kingdom exemplars point the way: subcellular fractionation and flux analysis resolve fast, compartment-locked control, often dictated by metabolite levels rather than enzyme abundance [536,537]. Physicochemical constraints and receptor transport further complicate KYNA partitioning, while KMO also governs mitochondrial morphology independent of catalysis [538,539]. Closing these blind spots will require synchronized sampling, stable-isotope fluxomics, spatial metabolomics, and organelle-targeted assays integrated across compartments and time.

A credible standardization roadmap rests on shared materials, shared methods, and shared language. First, anchor quantification with pooled multi-matrix reference materials, isotopologue-rich internal standards, and external proficiency testing to harmonize calibration and batch correction across sites [504,540]. Second, codify cross-lab standard operating procedures (SOPs) that cover pre-analytics, extraction, chromatography, ionization, and QC frequency, drawing on best-practice tutorials and ultra-high-performance liquid chromatography (UHPLC)–high-resolution mass spectrometry (HRMS) guidance to minimize matrix effects and maximize metabolome coverage [541,542]. Third, mandate harmonized reporting: adopt community annotation levels, findable, accessible, interoperable, and reusable (FAIR) data deposition, and transparent identification criteria with library spectra, retention-time locking, and predictive models for MS1 features [316,543]. Open, referenceable workflows should be executable end-to-end and versioned in platforms such as MetaboAnalystR and Workflow4Metabolomics, with simulated and real benchmark datasets for software validation and inter-laboratory comparability [544,545]. Finally, extend standardization to organelle-resolved assays using immunopurified mitochondria and absolute quantification, ensuring that pathway claims translate across compartments and cohorts [546,547].

Longitudinal, multi-layer cohorts can convert metabolomic snapshots into mechanistically anchored, prognostic readouts of mitochondrial health [548,549]. Population platforms such as the translational metabolic cohort study (TMCS) and consortium of metabolomics studies (COMETS) already pair deep phenotyping with serial biospecimens, enabling stable-isotope sub-studies and genetic instruments to infer directionality between metabolite shifts and organelle function [548,550]. Coupling these frameworks to targeted mitochondrial phenotyping—multiplexed respiratory flux assays, immune-cell subtype profiling, and organelle-resolved metabolomics—links pathway panels to actionable defects in energy transfer, biogenesis, and redox control [466]. Prognostic models in sepsis, diabetic kidney disease, glioma, and mitochondrial disorders illustrate translational yield when pathway signatures are integrated with outcomes and imaging [551].

Genomic and microbiome layers further de-risk translation by prioritizing causal metabolites, resolving ancestry-specific effects, and exposing host–microbe axes that shape mitochondrial phenotypes [93,552]. Systems proteomics in liver and cross-species maps of mitochondrial gene function provide mechanistic scaffolds to interpret cohort signals and nominate interventions [553,554]. The immediate agenda is practical: harmonize pre-analytics, embed reference materials, schedule repeated sampling, and co-capture mitochondrial function with metabolomics at scale [555]. Such integrated phenotyping will upgrade risk prediction, clarify therapeutic targets, and shorten the path from discovery to clinic [466,556].

Medicinal chemistry around KYNA is poised to pivot from blunt modulation to receptor-precise control [557]. Decades of structure–activity work has already yielded high-affinity glycine site antagonists at NMDA-Rs and clarified competitive versus noncompetitive mechanisms, setting a template for selective central nervous system probes [228,557]. Parallel advances nominate GPR35 as a mitochondrial-proximal target for ischemic protection and inflammasome control, motivating small molecules with tuned efficacy and residence at this GPCR [16,22]. AhR-active KYN scaffolds expand the chemical space for immunometabolic intervention, while a critical appraisal of purported α7 nicotinic activity focuses prioritization on validated targets [223,558]. Next steps should embrace biased agonism: leverage GPCR structural insights, cryptic allosteric pockets, and pathway-selective screening to favor mitochondrial Ca^2+^ restraint and anti-inflammatory outputs over broad immunosuppression [559,560]. Integration with enzyme-directed KYN-pathway modulators will enable orthogonal control of flux and signaling, improving the therapeutic index in neurodegeneration and oncology [110,561].

With the mechanistic map and measurement blueprint established, the translational question becomes operational: which lever is druggable, what mitochondrial endpoint should move, and what assay can verify it in patients. This section distills ligand and study design principles that follow directly from the receptor and QA to NAD^+^ architecture developed earlier.

## 6. Conclusions

This review converges on a simple piece of logic with wide reach: KYN metabolic flux, receptor signaling, and TCA cycle control co-determine mitochondrial resilience, redox poise, and immune tone. The authors integrate receptor biology with NAD^+^ economics and analytics, showing how KYNA–GPR35, AhR, and NMDA interfaces can be read alongside unified LC–MS panels [14,562]. The practical output is a mechanistic map that predicts mitochondrial endpoints from receptor and flux states, paired with an assay blueprint that can test those predictions in clinical cohorts. The take-home message is pragmatic. Mechanism and measurement must travel together to turn associative metabolomics into causal, intervention-ready biology. Three priorities emerge. First, clinically validated, harmonized assays that quantify KYN and TCA intermediates in single runs across matrices, with isotope-labeled standards and cross-lab QC. Second, causal trials that pair receptor-selective ligands or enzyme modulators with mitochondrial endpoints such as ATP preservation, ΔΨm stability, and mitophagy readouts. Third, longitudinal, compartment-aware cohorts linking pathway state to outcomes in neurology, psychiatry, cardiometabolic disease, and oncology [49]. These steps will de-risk translation, refine patient selection, and enable adaptive designs that use pathway ratios as eligibility and pharmacodynamic markers [63,66]. The authors’ comprehensive synthesis underscores a tractable path from circuits to clinics: receptor-selective KYNA analogs, de novo NAD^+^ support, and standardized analytics. Uncertainties remain around temporal dynamics, tissue specificity, and receptor–mitochondria causality in vivo [224]. Future work should integrate isotope tracing, organelle profiling, and receptor bias pharmacology within harmonized workflows. With these elements aligned, mechanistic precision can mature into durable therapies and decision-grade biomarkers.

## Figures and Tables

**Table 1:** How this review differs from prior review themes in the KYN pathway literature.

Prior Review Theme (Typical Emphasis)	What is Usually Well Covered	What is Commonly Underdeveloped	What This Review Adds	References
Neurology and psychiatry	Metabolite patterns, QA and KYNA balance, clinical associations	Receptor proximal steps that explain how signals reach mitochondria	Receptor to mitochondria causal chain with ATP, Ca^2+^, mitophagy, and inflammasome endpoints	[50,92]
NAD^+^ and aging biology	Quinolinate to NAD^+^ logic, sirtuins, redox and longevity framing	How receptor pharmacology reshapes NAD^+^ economics in specific tissues	QA to NAD^+^ to TCA integration tied to mitochondrial control nodes	[93,94]
Immunometabolism and oncology	IDO and TDO tolerance circuits, AhR-mediated immune programming	Mitochondrial mechanism often implicit rather than specified	Organellar mechanisms positioned as selectable therapeutic levers	[47,95]
Analytical and metabolomics reviews	KYN panels, targeted LC-MS methods, matrix effects	Co-measurement of KYN plus TCA plus NAD relevant nodes in one clinical run	Analytics for clinic workflows with single-run design choices and harmonization checkpoints	[96,97]

AhR, aryl hydrocarbon receptor; ATP, adenosine triphosphate; Ca^2+^, calcium ion; IDO, indoleamine 2,3-dioxygenase; KYN, kynurenine; KYNA, kynurenic acid; LC-MS, liquid chromatography–mass spectrometry; NAD^+^, nicotinamide adenine dinucleotide; QA, quinolinic acid; TCA, tricarboxylic acid; TDO, tryptophan 2,3-dioxygenase.

**Table 3:** Integration points linking the KYN metabolism to the TCA cycle and immune signaling. Concise map of biochemical “nodes” where KYN-pathway flux or signaling intersects mitochondrial control, NAD^+^/NADH balance, anaplerosis, and immune/ROS outcomes.

Node (e.g., QA → NAD^+^)	Enzymes/Regulators	Directionality	Impact on NAD^+^/NADH or Anaplerosis	Immune/ROS Consequence	Evidence Class	References
QA → NAD^+^	QPRT → NMNAT → NADS; modulation by aging/inflammation	KYN → NAD^+^ biogenesis → ETC	Increases cellular/matrix NAD(H); sustains complex I oxidation and O_2_ consumption	Supports macrophage respiration; QPRT loss ↓NAD^+^, ↑injury/ROS; neuroinflammation requires conversion for SIRT activity	Mechanistic (cells/animals), clinical/genetic	[14,42,230]
KYNA → GPR35 → mitochondrial nodes (incl. MAS)	KATs (KYNA production), GPR35; MAS components	KYNs ligands → receptor → mitochondria/TCA	Tunes ETC throughput and shuttle-coupled redox set-points	Sets immune tone; receptor-proximal control of inflammatory signaling	Mechanistic receptor signaling	[16,22,132]
IDO1/TDO2 rate-setting → α-KG availability	IDO1, TDO2; IFN, hypoxia, nutrient status	Immune cues → KYNs flux → TCA carbon	Pulls carbon from Trp; constrains or supports α-KG–linked anaplerosis	Biases tolerance vs. activation; conditions efficacy of IDOs/TDO blockade	Multi-omic/tumor-inflammation frameworks	[26,223,224]
MCART1-mediated NAD^+^ import (cytosol → matrix)	MCART1 (SLC25 family)	Cytosol → mitochondria	Maintains matrix NAD^+^ for dehydrogenases; prevents collapse of OXPHOS	Preserves respiratory control; supports T-cell effector programs	Mechanistic (transport/respiration)	[231,232,233]
MDH2 → oxaloacetate restraint of Complex II	MDH2; OAA	TCA intermediate → ETC modulation	Re-routes electron flow; dynamically resets NAD^+^/NADH coupling	Shapes graded ROS signaling downstream of ratio	Mechanistic ETC control	[219,220,234]
NNT couples NADH ↔ NADPH demand	Nicotinamide nucleotide transhydrogenase (NNT)	Matrix redox coupling	Balances NADH oxidation with NADPH generation; stabilizes redox poise	Supports antioxidant defenses; buffers ROS during substrate shifts	Mechanistic redox coupling	[235,236,237]
ME1 (malic enzyme-1): malate → pyruvate (NADPH)	ME1	Cytosol anaplerosis ↔ redox	Raises NADPH; protects glutathione/NADPH buffering	Reinforces antioxidant capacity; feeds redox-encoded signaling	Mechanistic redox metabolism	[238,239]
Serine catabolism → NADH accumulation when respiration stalls	One-carbon/serine axis	Amino acid metabolism → redox	Builds cytosolic/mitochondrial NADH when ETC is limited; throttles biosynthesis	Constrains proliferative programs under low respiration	Mechanistic metabolic control	[2,9,240]
SDH lesion → alternative aspartate synthesis (matrix NAD^+^/NADH-dependent)	SDH/Complex II context; aspartate pathways	ETC defect → rerouted biosynthesis	Forces aspartate generation routes that depend on matrix NAD^+^/NADH	Salvages growth despite impaired cycling	Mechanistic pathology	[241,242,243]
De novo NAD^+^ from KYNs supports macrophage respiration	QPRT→NMNAT→NADS; macrophage programs	KYNs → NAD^+^ → OXPHOS	Expands NAD(H) pool; sustains respiratory control across tissues	Coordinates systemic redox communication; tunes inflammatory effectors	Mechanistic + systems	[14,42,244]
Type I IFN → IDH inhibition → citrate/α-KG ratio shift	IDH1/IDH2; Type I IFN	Immune signal → TCA wiring → KYNs context	Alters NADPH generation/redox milieu that licenses IDOs/TDO activity	Reprograms Trp catabolism vs. defense state	Mechanistic immunometabolism	[26,221,234]
M1 macrophage “IDH break” → α-KG drop	IDH node; network integration	Polarization cue → TCA fragmentation	↓Anaplerosis; altered NADPH; redox favoring effector programs and IDO induction	Heightened inflammatory activation	Network/multi-omic	[234,245]
Sirtuin activity sustained by quinolinate-derived NAD^+^	SIRTs; QPRT/NMNAT/NADS	KYNs → NAD^+^ → sirtuin deacylases	Preserves mitochondrial protein deacylation and efficiency	Supports neuronal viability; mitigates inflammatory stress	Mechanistic neuroinflammation	[14,25,94]
Ischemia–reperfusion diversion away from quinolinate	Pathway branch choice; NAD^+^ augmentation	Stress → KYN branch → NAD^+^	NAD^+^ depletion when diverted; restoration rescues antioxidant capacity	Less oxidative injury with NAD^+^ repletion	Mechanistic/therapeutic modulation	[22,31,43]
UMPS bypass completing NAD^+^ synthesis when canonical steps fail	UMPS (bypass), salvage enzymes	Engineered/alternative route → NAD^+^	Raises total NAD(H) when QPRT or steps are compromised	Enhances ETC throughput in designed systems	Engineering proof-of-principle	[246,247,248]
Quinolinate/NAM rise tracks mitochondrial work/biogenesis	Systemic KYNs NAD^+^ salvage	Workload/biogenesis → KYNs output	Correlated elevation of circulating quinolinate & nicotinamide with ETC demand	Links tissue respiratory programs to systemic KYN tone	Integrative physiology	[94,249,250]
Ubiquinol (CoQH_2_) oxidation requirement beyond NAD^+^ regeneration	ETC Complex III/CoQ cycle	ETC constraint → metabolic outcome	NAD^+^ repletion alone insufficient if CoQ oxidation is limited	Governs tumor growth constraints; bioenergetic bottleneck	Mechanistic tumor bioenergetics	[104,219,220]
LKB1 programs & thioredoxin circuits sculpt NADH turnover	LKB1, TRX/thioredoxin	Kinase/antioxidant systems → NADH flux	Adjusts NADH oxidation and chromatin-linked NAD^+^ usage	Sets T-cell effector capacity	Mechanistic immune control	[251,252,253]

α-KG, alpha-ketoglutarate; CoQH_2_, ubiquinol; ETC, electron transport chain; GPR35, G protein-coupled receptor 35; IDO, indoleamine 2,3-dioxygenase; IDO1, indoleamine 2,3-dioxygenase 1; IDH, isocitrate dehydrogenase; IFN, interferon; KAT, kynurenine aminotransferase; KYN, kynurenine; KYNA, kynurenic acid; LKB1, liver kinase B1; MAS, malate–aspartate shuttle; MCART1, mitochondrial carrier of NAD^+^ transporter 1; MDH2, malate dehydrogenase 2; ME1, malic enzyme 1; NAM, nicotinamide; NADS, NAD^+^ synthetase; NADH, nicotinamide adenine dinucleotide (reduced form); NADPH, nicotinamide adenine dinucleotide phosphate (reduced form); NMNAT, nicotinamide mononucleotide adenylyltransferase; NNT, nicotinamide nucleotide transhydrogenase; OAA, oxaloacetate; OXPHOS, oxidative phosphorylation; QA, quinolinic acid; QPRT, quinolinate phosphoribosyltransferase; ROS, reactive oxygen species; SDH, succinate dehydrogenase; SIRT, sirtuin; SLC25, solute carrier family 25; TCA, tricarboxylic acid cycle; TDO, tryptophan 2,3-dioxygenase; Trp, tryptophan; TRX, thioredoxin; UMPS, uridine monophosphate synthase.

**Table 4:** Clinical and preclinical patterns connecting KYN metabolism–TCA crosstalk to disease phenotypes. Summary of disease-domain patterns linking KYN-pathway metabolites to mitochondrial phenotypes and clinical/functional readouts. Study type/size reflects the level of evidence stated in the text (meta-analyses/cohorts, biomarker studies, preclinical models, early clinical combination strategies) without inventing sample counts.

Indication (Neurodegeneration/Psychiatry/Oncology)	Metabolite Signature KYNA, QA, KYN, 3-HK	Mitochondrial Phenotype	Clinical/Functional Readouts	Study Type/Size	References
Neurodegeneration	KYNA ↓, QA ↑; KYN context-dependent; 3-HK not primary in text	NMDA-driven ROS; depressed respiratory capacity; feed-forward bioenergetic failure and inflammation	Higher QA:KYNA ratios track tau/amyloid burden, neuronal dysfunction, faster progression	Clinical & biomarker studies; aging/disease cohorts; preclinical mechanistic work	[51,52,80]
Neurodegeneration (therapeutic angle)	Shift flux away from QA; raise KYNA (e.g., KMO modulation)	Mitochondrial stabilization; restored antioxidant defenses (incl. Nrf2 signaling)	Slower neurodegeneration trajectory; reduced excitotoxic stress	Preclinical + translational strategy proposals; early phase targeting concepts	[3,8,207]
Psychiatry	Trp ↓, KYN ↓ (cohorts/meta-analyses); QA favored under immune activation; KYNA/KYN/QA show state-dependent oscillations; 3-HK not emphasized	Immune-bioenergetic coupling; mitochondrial/synaptic function shifts; serotonin depression when KYN metabolism is upshifted	Mood, psychosis, cognitive deficits; moderate blood–brain metabolite concordance indexing symptom burden/progression	Meta-analyses and multi-cohort studies; biomarker cohorts; mechanistic frameworks	[54,75,92]
Psychiatry (therapeutic angle)	Enzyme/flux control targeting; microbiome-sensitive modulators	Rebalancing KYNs–TCA redox and neurotransmission coupling	Symptom modulation and progression tracking via peripheral KYNs panels	Translational strategies; trial-readout integration (sizes vary)	[49,50,63]
Oncology (tumor immune escape)	KYN ↑ via IDO1/TDO2 → AhR activation; downstream KMO/KYNU shape invasive traits; KYNA/QA context-specific	Bioenergetic rewiring supporting growth; TME-conditioned redox	Treg expansion, CD8^+^ exhaustion; metastatic behavior, stromal crosstalk, chemoresistance	Clinical experience with IDOs/TDO blockade; preclinical tumor models; biomarker studies	[223,224,259]
Oncology (combination therapy)	KYN depletion (KYNU depots); AhR attenuation (e.g., small molecules)	Reprogrammed mitochondrial/immune metabolism; macrophage repolarization (e.g., GPX4–KYNU axis)	Synergy with PD-1 blockade; dismantling suppressive niches; overcoming resistance	Preclinical synergy studies; early translational combinations; emerging clinical strategies	[74,223,244]

3-HK, 3-hydroxykynurenine; AhR, aryl hydrocarbon receptor; CD8^+^, cluster of differentiation 8 positive; GPX4, glutathione peroxidase 4; IDO1, indoleamine 2,3-dioxygenase 1; KMO, kynurenine 3-monooxygenase; KYN, kynurenine; KYNA, kynurenic acid; KYNU, kynureninase; Nrf2, nuclear factor erythroid 2–related factor 2; PD-1, programmed cell death protein 1; QA, quinolinic acid; ROS, reactive oxygen species; TCA, tricarboxylic acid cycle; TDO2, tryptophan 2,3-dioxygenase 2; TME, tumor microenvironment; Trp, tryptophan; Treg, regulatory T cell.

**Table 5:** LC-MS/MS bioanalytical validation criteria for the simultaneous measurement of KYNs and TCA cycle metabolites.

Parameter	Acceptance Threshold	Notes/Mitigations	References
Calibration model	R^2^ ≥ 0.98; back-calculated concentrations within ±15% (±20% at LLOQ)	Use matrix-matched standards; weighted (1/x or 1/x^2^) regression for wide dynamic ranges; verify linearity and curvature.	[468]
Linearity/Dynamic range	At least 6–8 calibration levels; deviation ≤ 15% (≤20% at LLOQ)	For TCA acids with steep response, consider quadratic fit; re-optimize injection volume to avoid saturation.	[469]
LLOQ/ULOQ	Signal ≥ 5 × noise; precision ≤ 20%; accuracy 80–120%	Confirm LLOQ in actual matrix (plasma/CSF). Check for ion suppression at LLOQ.	[470]
Carryover	<20% of LLOQ signal and <5% of IS signal in blank after highest standard	Insert strong wash; consider needle wash with ACN/MeOH/water + 0.1% FA; extend gradient re-equilibration.	[471]
Matrix effect (ME)	IS-normalized ME CV ≤ 15% across ≥ 6 donors	Perform post-column infusion; evaluate ME at low/mid/high QC; use isotopologues matched by polarity and retention.	[438,471,472]
Extraction recovery	80–120% with CV ≤ 15%	Compare pre-spike vs. post-spike; optimize precipitation solvent and pH; avoid ion-pairing agents in precipitant.	[438,473]
Precision—intra-day (repeatability)	CV ≤ 15% (≤20% at LLOQ)	Analyze ≥ 5 replicates each QC level; monitor retention shifts and ion ratio stability.	[470]
Precision—inter-day (reproducibility)	CV ≤ 15%	Include replicate QCs on ≥3 days; reinject archived QCs to detect long-term drift.	[469]
Accuracy	85–115% (80–120% at LLOQ)	Compare to spiked reference material; evaluate both absolute and IS-normalized values.	[468]
Short-term autosampler stability	≤15% change over 4–24 h at 4–8 °C	KYNs TCAs, may degrade—use immediate analysis or stabilizing additives.	[474]
Freeze–thaw stability	≤15% change after 3 cycles	Avoid repeated thawing; aliquot samples; confirm TCA acids stability separately.	[471]
Long-term stability (−70 °C)	≤15% deviation	Validate ≥ 1–3 months	[475]
Processed sample stability	≤15% after 6–12 h in autosampler	For unstable analytes use rapid acquisition.	[476]
System suitability	Retention time shift <0.2–0.3 min; IS area CV ≤ 10%; ion ratio within ±20%	Verify before each batch; monitor column pressure, peak shape, and polarity switching efficiency.	[477]
QC frequency per batch	≥3 levels (LQC/MQC/HQC); QCs at start, every 10–15 samples, and end	Large cohorts: insert pooled QC every 10–12 injections.	[470]
Batch acceptance criteria	≥67% of all QCs and ≥50% at each level must be within ±15% (±20% at LLOQ)	If failure: investigate ME shifts, IS suppression, column fouling, or calibration instability.	[469]
Inter-batch comparability	QC CV ≤ 15% across batches	Use pooled QC and IS-normalized drift correction.	[478]

R^2^: coefficient of determination; LLOQ: lower limit of quantification; KYNs: kynurenines; TCA: tricarboxylic acid cycle; ULOQ: upper limit of quantification; CSF: cerebrospinal fluid; ACN: acetonitrile; MeOH: methanol; FA: formic acid; ME: matrix effect; IS: internal standard; QC: quality control; CV: coefficient of variation; LQC/MQC/HQC low-/medium-/high-quality control.

**Table 6:** Proposed comprehensive LC–MS/MS panels covering the KYN pathway and TCA cycle for clinical and translational studies. Targeted LC–MS/MS panel covering key metabolites of the KYN pathway and TCA cycle in clinically relevant biological matrices. Analytes were selected based on biological relevance and analytical feasibility in targeted workflows. Recommended SIL-IS, chromatographic separation modes, and optimal ESI polarity are indicated. HILIC, including mixed-mode variants where appropriate, is suggested to enable retention of highly polar metabolites. Key stability considerations highlight compounds requiring particular attention during sample handling and processing.

Analyte	Matrix	Internal Standard	Key Stability Issue	Clinical Relevance	Chromatography	Polarity	Reference
Trp	Plasma, serum	d_5_-Tryptophan	Stable; affected by hemolysis	Substrate availability; KYN/TRP ratio for IDO/TDO activity	HILIC compatible	ESI^+^	[89]
KYN	Plasma, serum	d_4_-Kynurenine	Generally stable; protein binding may affect recovery	Central branch-point of KYN metabolism; immune activation marker	HILIC compatible	ESI^+^	[50]
KYNA	Plasma, serum, CSF	d_5_-Kynurenic acid	Light sensitivity; adsorption at low concentrations	NMDA, GPR35 signaling; neuroprotective and immunomodulatory roles	HILIC compatible	ESI^+^	[206]
3-HK	Plasma	d_3_-3-Hydroxy-kynurenine	Oxidation-prone; requires antioxidant stabilization	Pro-oxidant stress marker; neurotoxicity and redox imbalance	HILIC compatible	ESI^+^	[89]
AA	Plasma, urine	d_4_-Anthranilic acid	Generally stable	Alternative branch activity; inflammatory pathway balance	HILIC compatible	ESI^+^	[483]
3-HAA	Plasma	d_3_-3-Hydroxy-anthranilic acid	Oxidation-sensitive; light exposure	Immunomodulation; redox-active intermediate	HILIC compatible	ESI^+^	[484]
XA	Plasma, urine	d_4_-Xanthurenic acid	Moderate stability; pH-dependent	Metabolic dysregulation; diabetes and mitochondrial stress associations	HILIC compatible	ESI^+^	[485]
QA	Plasma, urine, CSF	d_3_-Quinolinic acid	Freeze–thaw sensitive; oxidative degradation	Excitotoxicity; NAD^+^ precursor flux; neuroinflammation	HILIC compatible	ESI^+^	[89]
PA	Plasma, urine	d_4_-Picolinic acid	Generally stable	Metal chelation; immune modulation	HILIC compatible	ESI^+^	[484]
Citrate	Plasma	^13^C_2_-citrate	Moderate instability; delayed processing sensitive	Mitochondrial entry point; energy metabolism proxy	HILIC compatible	ESI^−^	[234]
Isocitrate	Plasma	^13^C_2_-citrate	Isomerization risk; low abundance	TCA flux regulation; redox coupling	HILIC compatible	ESI^−^	[234]
α-Ketoglutarate	Plasma	^13^C_5_-α-ketoglutarate	Rapid degradation; requires fast quenching	Central metabolic node; immunometabolic signaling	HILIC compatible	ESI^−^	[234]
Succinate	Plasma	d_4_-Succinate	Rapid post-sampling increase	Inflammatory signaling; mitochondrial dysfunction	HILIC compatible	ESI^−^	[234]
Fumarate	Plasma	^13^C_4_-Fumarate	Generally stable	Oncometabolic and hypoxic signaling	HILIC compatible	ESI^−^	[234]
Malate	Plasma	^13^C_4_-malate	Moderate instability	NADH/NAD^+^ coupling; mitochondrial redox state	HILIC compatible	ESI^−^	[234]

3-HAA, 3-hydroxyanthranilic acid; 3-HK, 3-hydroxykynurenine; AA, anthranilic acid; CSF, cerebrospinal fluid; ESI, electrospray ionization; ESI^+^, positive electrospray ionization; ESI^−^, negative electrospray ionization; GPR35, G protein-coupled receptor 35; HILIC, hydrophilic interaction liquid chromatography; IDO, indoleamine 2,3-dioxygenase; KYN, kynurenine; KYNA, kynurenic acid; LC–MS/MS, liquid chromatography–tandem mass spectrometry; NAD^+^, nicotinamide adenine dinucleotide; NMDA, N-methyl-D-aspartate; PA, picolinic acid; QA, quinolinic acid; SIL-IS, stable isotope-labeled internal standards; TCA, tricarboxylic acid cycle; TDO, tryptophan 2,3-dioxygenase; TRP, tryptophan; XA, xanthurenic acid.

## Data Availability

No new data were created or analyzed in this study.

## Abbreviations

The following abbreviations are used in this manuscript:

α7nAChR	nicotinic acetylcholine receptors containing α7 subunits
ACMSD	alpha-amino-beta-carboxymuconate-epsilon-semialdehyde decarboxylase
AhR	aryl hydrocarbon receptor
AKT	protein kinase B
AMPK	AMP-activated protein kinase
ATP	adenosine triphosphate
ATPIF1	ATP synthase inhibitory factor 1
BNIP3	BCL2 interacting protein 3
cAMP	cyclic adenosine monophosphate
CD98	cluster of differentiation 98
CLSI	clinical and laboratory standards institute
COMETS	consortium of metabolomics studies
CSF	cerebrospinal fluid
ERK	extracellular signal-regulated kinase
ETC	electron transport chain
FAIR	findable, accessible, interoperable, and reusable
GPR35	G protein-coupled receptor 35
GPX4	glutathione peroxidase 4
HIF-1α	hypoxia-inducible factor 1 alpha
HILIC	hydrophilic interaction liquid chromatography
HRMS	high-resolution mass spectrometry
IDO	indoleamine 2,3-dioxygenase
IDO1	indoleamine 2,3-dioxygenase 1
IDO2	indoleamine 2,3-dioxygenase 2
KATs	kynurenine aminotransferases
KMO	kynurenine 3-monooxygenase
KYN	Kynurenine
KYNA	kynurenic acid
KYNU	Kynureninase
LC–MS	liquid chromatography–mass spectrometry
LLOQ	lower limit of quantitation
MCART1	mitochondrial carrier transporting NAD^+^
MDH2	malate dehydrogenase 2
mPTP	mitochondrial permeability transition pore
NAD^+^	nicotinamide adenine dinucleotide (oxidized form)
NADH	nicotinamide adenine dinucleotide (reduced form)
NADPH	nicotinamide adenine dinucleotide phosphate (reduced form)
NF-κB	nuclear factor kappa-light-chain-enhancer of activated b cells
NMDA	N-methyl-D-aspartate
NMDA-R	N-methyl-D-aspartate receptor
NRF1	nuclear respiratory factor 1
NRF2	nuclear factor erythroid 2–related factor 2
PD-1	programmed cell death protein 1
PI3K	phosphoinositide 3-kinase
QC	quality control
QA	quinolinic acid
Rho	ras homolog family protein
ROS	reactive oxygen species
SAR	structure–activity relationship
SDH	succinate dehydrogenase
SOP	standard operating procedure
TCA	tricarboxylic acid
TDO	tryptophan 2,3-dioxygenase
TMCS	translational metabolic cohort study
TME	tumor microenvironment
Trp	Tryptophan
UHPLC	ultra-high-performance liquid chromatography
